# The roles, challenges, and merits of the p value

**DOI:** 10.1016/j.patter.2023.100878

**Published:** 2023-12-08

**Authors:** Oliver Y. Chén, Julien S. Bodelet, Raúl G. Saraiva, Huy Phan, Junrui Di, Guy Nagels, Tom Schwantje, Hengyi Cao, Jiangtao Gou, Jenna M. Reinen, Bin Xiong, Bangdong Zhi, Xiaojun Wang, Maarten de Vos

**Affiliations:** 1 Département Médecine de Laboratoire et Pathologie, Centre Hospitalier Universitaire Vaudois, Lausanne, Switzerland; 2 Faculté de Biologie et de Médecine, Université de Lausanne, Lausanne, Switzerland; 3 Department of Molecular Microbiology and Immunology, Johns Hopkins University, Baltimore, MD, USA; 4 Department of Computer Science, Queen Mary University of London, London, UK; 5 Department of Biostatistics, Johns Hopkins University, Baltimore, MD, USA; 6 St. Edmund Hall, University of Oxford, Oxford, UK; 7 Department of Neurology, Universitair Ziekenhuis Brussel, Vrije Universiteit Brussel, Jette, Belgium; 8 Department of Economics, University of Oxford, Oxford, UK; 9 Institute of Behavioral Science, Feinstein Institutes for Medical Research, Manhasset, NY, USA; 10 Division of Psychiatry Research, Zucker Hillside Hospital, Glen Oaks, NY, USA; 11 Department of Mathematics and Statistics, Villanova University, Villanova, PA, USA; 12 IBM Thomas J. Watson Research Center, Yorktown Heights, NY, USA; 13 Department of Statistics, Northwestern University, Evanston, IL, USA; 14 School of Business, University of Bristol, Bristol, UK; 15 Birmingham Business School, University of Birmingham, Birmingham, UK; 16 Faculty of Engineering Science, KU Leuven, Leuven, Belgium; 17 Faculty of Medicine, KU Leuven, Leuven, Belgium

## Abstract

Since the 18th century, the p value has been an important part of hypothesis-based scientific investigation. As statistical and data science engines accelerate, questions emerge: to what extent are scientific discoveries based on p values reliable and reproducible? Should one adjust the significance level or find alternatives for the p value? Inspired by these questions and everlasting attempts to address them, here, we provide a systematic examination of the p value from its roles and merits to its misuses and misinterpretations. For the latter, we summarize modest recommendations to handle them. In parallel, we present the Bayesian alternatives for seeking evidence and discuss the pooling of p values from multiple studies and datasets. Overall, we argue that the p value and hypothesis testing form a useful probabilistic decision-making mechanism, facilitating causal inference, feature selection, and predictive modeling, but that the interpretation of the p value must be contextual, considering the scientific question, experimental design, and statistical principles.

## The bigger picture

The discussions of hypothesis testing and the p value are enduring. They are, however, done in relative isolation; theorists inspect them using mathematical arguments, and applied scientists scrutinize them via experimental intuition. Most are aware that the interpretation of the p value needs to be contextual, but what does “contextual” mean? Here, linking examples and equations, we present a relatively comprehensive inquiry into the foundations, merits, and challenges of hypothesis testing and the p value: why they are useful and when negligence may occur. We build presentations from relatively simple history, philosophy, and cases to slightly complex statistical reasoning. We endeavor to make our language accessible and stories complementary to a broad audience; some apply those instruments frequently, some aspire to develop new methods, and perhaps all hope to one day find a cogent way to translate patterns from data into knowledge.

## Teaser

In this review, the authors discuss the roles, challenges, and merits of the p value in hypothesis-based scientific inquiry and emphasize why the interpretation of the p value needs to be contextual. They then present arguments from causal inference, feature selection, and predictive modeling. The authors explore common misuses and misinterpretations and potential remedies and examine Bayesian alternatives, meta-analysis methods for combining several p values, and multiple comparison techniques. Real-world examples and detailed statistical arguments for audiences with different backgrounds are provided.

## Introduction

David Hume argued in *A Treatise of Human Nature* that “all knowledge degenerates into probability.”^1^ Probable inference is chief in guiding decisions.^2^^,^^3^^,^^4^ Sports fans make bets on the likelihood that a club will win the next game. Investors decide to buy or sell a stock based on how likely it is to go up or down. One chooses whether to bring an umbrella given the chance of rain. But what about scientists? Does probability guide scientific enquiries, and, if so, how?^5^

**Figure 1:**
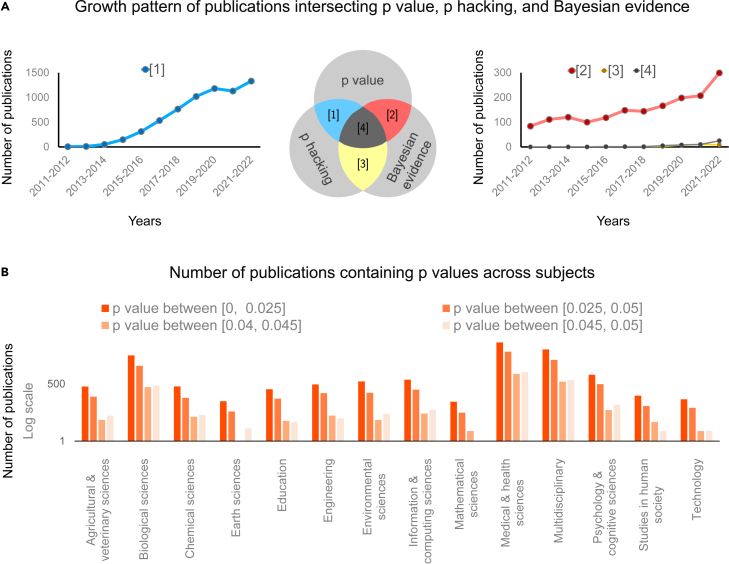
Recent trends of p value, *p hacking*, and Bayesian evidence in scientific studies (A) The growth pattern of the p value in the past decade. Recent years have witnessed a considerable increase in articles consisting of topics related to p value, *p hacking*, and Bayesian evidence. Particularly, articles that discuss both p value and *p hacking* as well as those that discuss both p value and Bayesian evidence have grown in an exponential-like trajectory. We used the following literature search strategy. We first define three sets of keywords: PV (p value), PH (*p hacking*), and BE (Bayesian evidence). We then define publications in [1]–[4] as PV ∩ PH ∖ BE, PV ∩ BE ∖ PH, PH ∩ BE ∖ PH, and PV ∩ PH ∩ BE, respectively. The search used the advanced search function provided by Google Scholar, where, for example, the query PV ∩ PH ∖ BE is equivalent to entering “p value; *p hacking* -Bayesian evidence.” (B) The distribution of the p values across academic disciplines. The p values are widely used in 14 common subjects, noticeably in biological sciences, medical and health sciences, multidisciplinary fields, and psychological and cognitive sciences. Across different subjects, smaller p values (those between 0 and 0.025) seem to be more commonly reported than larger (albeit significant at 0.05) counterparts. Data for plotting (B) are from Head et al.^6^

The p value-based hypothesis testing is a widely used principle in scientific decision-making. Text mining using 385,393 PubMed Central (PMC) articles from 1990–2015 identified 3,438,299 appearances of p values; that is, about nine p values per article.^7^ It has interested social scientists,^8^ philosophers,^9^ biomedical scientists,^10^ clinicians,^11^ and ecologists^12^ no less than statisticians (Figure 1). Yet, as a probabilistic statement underpinning decision-making, the p value has generated enduring debates.^13^^,^^14^^,^^15^^,^^16^^,^^17^^,^^18^^,^^19^^,^^20^ Central to these debates is its inconsistency and potential lack of credibility in providing evidence. To raise protection, scholars have suggested lowering the significance level from 0.05 to 0.01^21^ or 0.005.^22^^,^^23^ Others have asked whether the p value (and, therefore, the significance test) should be banned.^24^^,^^25^^,^^26^ The *Basic and Applied Social Psychology* (BASP) journal, at perhaps the extreme end, cast an editorial ban on the p value.^27^

The debates and ensuing responses have inspired us to have a thorough reflection on and discussion about the p value, from its origin and definition to its usefulness, misuses, and potential mitigations. Fortunately, we have access to a resourceful volume of past works on the p value in the fields of economics, statistics, biology, and philosophy. Standing on the shoulders of the pioneers, we make our addition.

We begin with a brief history of the p value. We then outline the roles the p value plays in scientific enquiries, including causal inference, feature selection, and predictive modeling. Next, we present its common misuses, misinterpretations, and potential treatments. Subsequently, we compare statistical significance and clinical relevance. In parallel, we examine Bayesian evidence and discuss the promises and challenges of pooling p values from multiple studies and datasets via meta-analysis and multiple comparisons. We conclude with a discussion. Through our explorations and discussions, we argue that the p value and hypothesis testing are useful devices for extracting evidence, but one needs to employ and interpret them in context, considering the scientific question, experimental design, model specification, statistical power, effect size, whether there is prior knowledge, and reproducibility.

### A brief history of the p value

#### The debut of the p value

John Arbuthnot performed perhaps the first significance testing (see page 40 in Heyde et al.^28^), although the origin of the hypothesis test and the p value is difficult to trace. Having observed that the number of males born in London exceeded the number of females for 82 consecutive years (1629–1710), Arbuthnot wanted to examine whether the birth rates of males and females were equal. He assumed two hypothetical individuals, A and B, where B claimed that “…every year there shall be born more Males than Females,” and A laid a hypothesis against B’s. He then argued that, if the birth rates were equal, then the probability of observing more male newborns for 82 consecutive years would be (0.5^82^).^29^ Based on this infinitely small likelihood, he concluded that the birth rates were not equal. It was a relatively simple sign test, but “the first example of reasoning about statistical significance”^30^ and “perhaps the first published report of a nonparametric test.”^31^

#### The rise of the p value

“Throughout the 19th century, hypothesis testing was carried out rather informally without a prespecified rejection level. It was roughly equivalent to calculating a (approximate) p value and rejecting the hypothesis if this value appeared to be sufficiently small.”^32^

Francis Edgeworth and Karl Pearson advanced the practice of significance tests during the late 19th and early 20th centuries. The former designed a test to compare means from two samples^33^ and introduced the concept of standard distance and a rejection rule (see chapter 13 in Spanos^34^). The latter introduced the chi-square test and calculated the tail probability (which he denoted as capital *P*) by integration.^35^ Edgeworth implicitly used the tail probability, or the p value, in his test, followed by Pearson’s formalization. It is, therefore, reasonable to credit them, in concert, with the very considerable contribution to establishing the concept of the p value.

The next milestone was made by R.A. Fisher. In his seminal work,^36^ Fisher argued that “the (critical) value for which p = 0.05, or 1 in 20, is 1.96 or nearly 2 (standard deviations); it is convenient to take this point as a limit in judging whether a deviation is to be considered significant or not. Deviations exceeding twice the standard deviation (under a standard normal distribution defined on R) are thus formally regarded as significant.” He also recast Pearson’s descriptive statistics into a model-based statistical induction, which changed the *ad hoc* approaches before him.^34^

**Figure 2:**
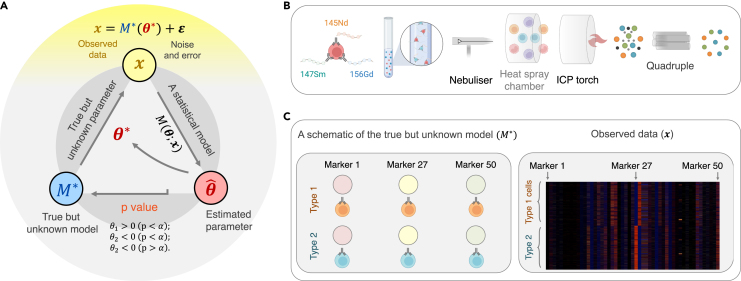
The fundamental goal of hypothesis testing in science (A) The triad of data-generating mechanism, observed data, and uncovering the true mechanism via hypothesis testing. One chief goal of scientific investigation is to understand the underlying (biological or physical) mechanism that gives rise to the observed data. When one has no or only preliminary knowledge about the mechanism $M^{*}$ and its parameters $\theta^{*}$, one hopes to learn about the mechanism and its parameters using observed data x, which are generated via $x=M^{*}\left(\theta^{*}\right)+\varepsilon$, where ε indicates noise and measurement errors. To do so, one proposes a model M with parameters θ (which, one hopes, approximate $M^{*}$ and $\theta^{*}$), and, given data x, obtains the estimated parameter $\hat{\theta }$. One then performs hypothesis tests to examine how close the estimated parameter may be to $\theta^{*}$. (B) The true but unclear data-generating mechanism. Using CyTOF (cytometry by time of flight) mass cytometry, rare earth metal isotopes are coupled to antibodies via a chelator tag, which is detected by a mass cytometer to quantitatively assess the concentrations of antibody-specific antigen present on a given cell. From left to right: cells are first incubated with a cocktail of metal isotope-labeled antibodies, washed to remove unbound antibodies, and then sprayed into droplets using a nebulizer. The droplets are dried in the heated spray chamber, allowing antibody-bound cells to individually enter the inductively coupled plasma (ICP) flame, resulting in instantaneous atomization of the cell into an ion cloud with its corresponding elemental composition. Elements found in normal biological samples with a mass of less than 80 atomic mass unit (AMU) are filtered out in the quadrupole, and the remaining rare earth metals coupled with specific antibodies are measured using a time-of-flight analyzer. (C) The true model and the observed data. Left: a schematic representation of the CyTOF model. Two new types of cells are marked by 50 different biomarkers. There exists a true data-generating mechanism $M^{*}$ driven by some parameter $\theta^{*}=\left(\theta_{1}^{*},\theta_{2}^{*},...,\theta_{50}^{*}\right)$, where $\theta_{i}^{*}$ determines whether the $i^{th}$ biomarker tags one cell type, both cell types, or neither (see text for details). We do not know about $M^{*}$ or $\theta^{*}$. Right: starting from the data, one proposes a statistical model to discover, via a hypothesis test, significant biomarkers that can distinguish the two cell types. Parts of (B) and (C) were drawn using BioRender.

To better understand p value-based decision-making, it is perhaps helpful to discern the fundamental goal of a hypothesis test and how the p value helps to address this goal. The fundamental goal of performing hypothesis testing is to derive evidence from the observed data to uncover the (underlying) mechanism that gives rise to the observed data (see Figure 2 and supplemental information for mathematical formulations). The mechanism can be biological or physical; uncovering the mechanism not only improves the description of the observed data but also discovers the biological or physical, and hence potentially causal, underpinning of the data.

Let us take an example. Consider 50 different isotopically pure rare earth metals attached to antibodies binding specific markers that are differentially expressed on two types of cells (type 1 and type 2). We want to know which of the biomarkers can distinguish the two types of cells. Suppose there are 100,000 cells (50,000 type 1 cells and 50,000 type 2 cells) and they are “stained” by all 50 biomarkers (a cell may attach to several biomarkers). The data are, therefore, a matrix of 100,000 rows and 50 columns, and each entry shows how much every biomarker is expressed on each cell. Let $x_{0}$ denote the data, a matrix of dimensionality 100,000 by 50.

Let $\theta^{*}=\left(\theta_{1}^{*},\theta_{2}^{*},...,\theta_{50}^{*}\right)$ denote the parameters that drive the true data-generating mechanism $M^{*}$. It is necessary to assume that such a data-generating mechanism exists and is generally consistent under similar experimental conditions and that one can derive new knowledge by uncovering the mechanism. In the above example, a true mechanism is a biological deposition so that the $i^{th}$ biomarker either binds type 1 cells and/or type 2 cells or does not bind (note that each cell type may need several different combinations of markers for specific identification). More concretely, suppose $\theta_{i}^{*}$ designates the mean difference between the expressions of the $i^{th}$ biomarker regarding two cell groups. But we do not know about $\theta^{*}$ and, therefore, do not know about the model driven by it (i.e., $M^{*}\left(\theta^{*}\right)$).

One way to learn about the unknown $\theta^{*}$ and the (biological or physical) mechanism $M^{*}$ is to propose a statistical model $M_{\theta }\left(x_{0}\right)$ that takes in data $x_{0}$ and puts out an estimated parameter $\hat{\theta }$. A good model renders a $\hat{\theta }$ that is close to $\theta^{*}$. But to determine how well the model performs, one needs to quantify how close $\hat{\theta }$ and $\theta^{*}$ are using some measure (metric). A distance metric may work, but it may be subject to its magnitude; for example, if the estimated distance from the Earth to the Sun is off by a few hundred kilometers, then one can say it is rather accurate, but if the estimated distance between your workplace and your home is off by a few hundred meters, it is less impressive. The p value, a probabilistic measure between 0 and 1, allows different individuals to quantify and compare the statistical significance of their results. To quantify a probabilistic measure, a useful way is to perform a hypothesis test: one first generates a (test) statistic from the data and then calculates the tail probability (the p value) to evaluate the strength of support for the hypothesis or lack thereof. Because the null-hypothesis statistical test (NHST) has been widely used in scientific studies (e.g., in biological studies,^37^ education,^38^ psychology,^39^^,^^40^ and social sciences^41^) and has been adopted by textbook writers, journal editors, and publishers,^39^^,^^42^ we use the NHST to develop our discussion.

### The definition of the p value

**Figure 3:**
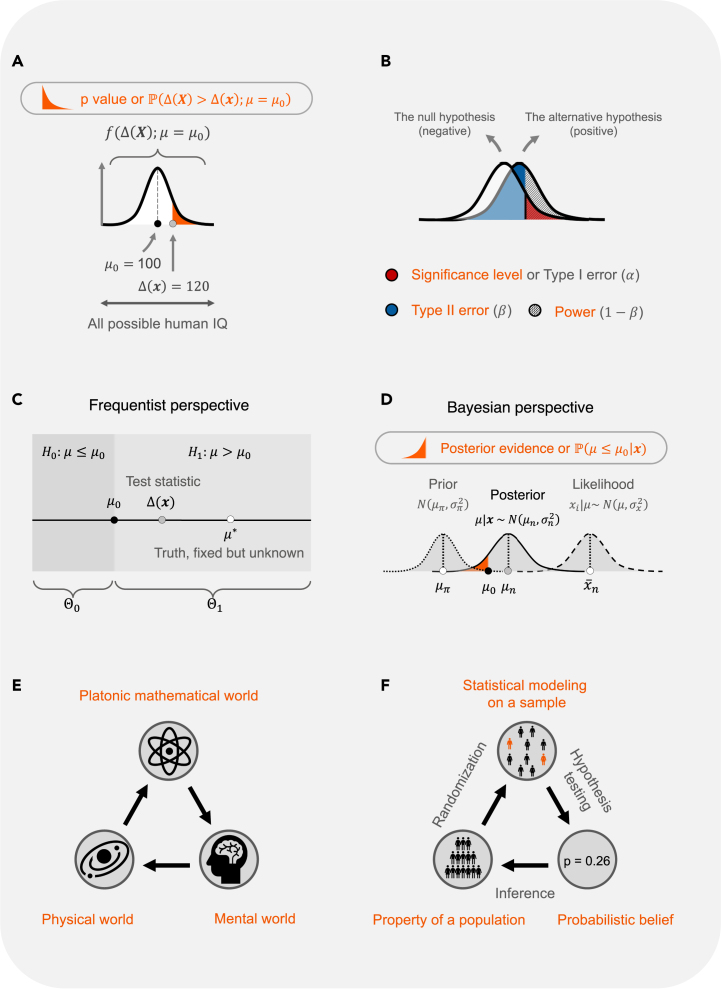
The p value and related concepts (A) Calculating the p value (see text for details). (B) Significance level (type I error), type II error, and power. The significance level (type I error or α) is a predetermined value (say 0.05), which quantifies the probability of observing extreme values given that the null hypothesis is true (red shades). The type II error (or β) quantifies the probability of failing to reject the null hypothesis given that the alternative hypothesis is true (blue shades). The power (or 1−β) quantifies the probability of rejecting the null hypothesis given that the alternative hypothesis is true (dashed shades). The value 1−α quantifies the probability of failing to reject the null hypothesis when it is true (represented by the white area, not completely shown, under the null hypothesis curve). (C) The frequentist perspective of the p value. In the frequentist view of hypothesis testing, the parameter is considered as an unknown constant rather than a random variable. (D) Bayesian perspective of evidence seeking. Suppose the prior knowledge weakly supports the null hypothesis $H_{0}:\mu \leq \mu_{0}$ (with a mean $\mu_{\pi }$ that sits slightly left of $\mu_{0}$), and the likelihood function has a center ${\bar{x}}_{n}$ that is far right of $\mu_{0}$. Then, the posterior mean $\mu_{n}$ is pulled, after seeing the data, in a direction rightward away from $\mu_{\pi }$ and toward $\mu_{0}$ and beyond; the farther the center of the likelihood function is from $\mu_{0}$ (namely, the more evidence the data provide against the null), the farther the posterior mean $\mu_{n}$ is pulled rightward away from $\mu_{0}$, and there is, therefore, stronger *a posteriori* evidence supporting the alternative hypothesis. (E) The three-world system—the physical world, the Platonic mathematical world, and the mental world—and our modification of it. The physical world represents the entire universe (from every chemical element to every individual) and contains properties that are not readily accessible to the observer. Some of these properties are governed by and/or can be explained using mathematical principles. The mathematical principles translate into (mental) understanding and form one’s perspective about the physical world. (F) The role of the p value in making scientific enquires. Consider an example where a clinician was making inquiries into the prevalence of a disease in a specific age group (i.e., a specific population). Suppose the clinician considered a null hypothesis where the prevalence was 10% (in the population). Because measuring the prevalence of a disease in a population was impractical, the clinician selected a random sample of 10 individuals from the population falling in that age group (left arrow) and found that two had the disease (top circle). The clinician then conducted a hypothesis test that generated a p value of 0.26 (right arrow) and used this to make inferences about the population (bottom arrow). Given the p value, the clinician concluded that there was not enough evidence (at a significance level of 0.05) from the sample that would reject the null hypothesis (made about the population).

Put simply, the p value is the tail probability calculated using a test statistic (see Figure 3A). To define it formally, let us use an example. A psychologist was interested in estimating the average fluid intelligence (Gf) in a specific age group. Suppose Gf follows a normal distribution, and we denote $X_{i}$ as the Gf score for an individual i∈{1,2,...}, then

$$\begin{array}{ccc} X_{i} & \begin{array}{c} \underset{∼}{i.i.d.} \end{array} & N\left(\mu ,\sigma^{2}\right) \end{array}$$

where i.i.d. means independent and identically distributed, $E\left(X_{i}\right)=\mu$, and $Var\left(X_{i}\right)=\sigma^{2}\;>0$.

Suppose there is no prior knowledge about the disease; the psychologist hypothesized that the average intelligence was less than or equal to 100 in that age group. That is, the psychologist hypothesized that the unobserved (but true, population) mean μ was less than or equal to $\mu_{0}$, where $\mu_{0}$ is set at 100; note that μ is a fixed value, not a random variable. This forms the null hypothesis $H_{0}:{\mu \leq \mu }_{0}$. In other words, the null hypothesis is true so long as the true parameter falls in the parameter space $Μ≔\left[0,\mu_{0}\right]$. The alternative hypothesis is that μ was greater than $\mu_{0}$, namely $H_{1}:{\mu =\mu }_{1}$, for any $\mu_{1}>\mu_{0}$.

Suppose now there is some prior knowledge supporting the null hypothesis $H_{0}:\mu \leq \mu_{0}$ (with a mean $\mu_{\pi }$ that sits slightly left of $\mu_{0}$), and the likelihood function has a center ${\bar{x}}_{n}$ that is far right from $\mu_{0}$ (see Figure 3D). Then, the posterior mean $\mu_{n}$ is pulled, after seeing the data, in a direction rightward away from $\mu_{\pi }$ and toward $\mu_{0}$ and beyond; the farther the center of the likelihood function is from $\mu_{0}$ (namely, the more evidence the data provide against the null), the farther the posterior mean $\mu_{n}$ is pulled rightward away from $\mu_{0}$, and there is, therefore, stronger *a posteriori* evidence supporting the alternative hypothesis. To avoid confusion, unless otherwise specified, in this paper we speak of p value in the frequentist sense; we will discuss Bayesian evidence under “Hypothesis test in the Bayesian realm.”

Now consider a null hypothesis *H_0_*: *μ* = 100. Suppose that one draws two samples and finds that the average Gf score from the first sample is 90 and that from the second sample is 110. This may yield a contradiction where the p values from samples 1 and 2 may each reject the null, but after combining the two samples, the p value may fail to reject the null. It is also possible that findings from the combined sample would still reject the null but the p value changes. The p value, therefore, depends on the sample data.

How, then, may the p value, a data-specific entity, be useful for scientific discovery, which requires a degree of universality? First, (scientific) universality does not mean a discovery holds for every situation and for every “experimental unit” (individual, plot, etc.). For example, the utility of penicillin does not suggest that it works on everyone or every infection (i.e., sample or data specific). The heart of data-driven scientific enquiry is to extract knowledge from the data via a statistical model (e.g., hypothesis testing). But to draw conclusions about the evidence and to raise gained knowledge to the rank of science, one needs to quantify the degree of trustworthiness (including reliability and reproducibility). The p value offers such a (probabilistic) quantification (about the belief) when there is little to no *a priori* knowledge (see “Hypothesis test in the Bayesian realm”).

**Figure 4:**
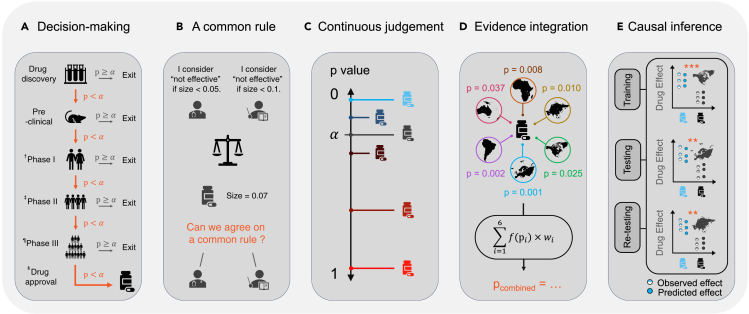
A few key useful roles of the p value From left to right: (A) It underpins a simple and clear decision-making system that has been accepted by broad scientific, clinical, and medical communities. ^†^Phase I is primarily aimed at safety and tolerability and, in a second order, on pharmacokinetics and pharmacodynamics. ^‡^In phase II, the study is most of the time not powered for a clinical endpoint but rather for a biomarker. ^¶^Phase III must indeed be significant. ^§^For drug approval, significance is important, but also safety issues and effect size. (B) It provides a common, and straightforward rule that guides multiple experimenters to evaluate and compare findings based on respective p values and a pre-agreed significance level. (C) It evaluates the outcomes of a test on a continuous scale. (D) It allows integrating results from multiple studies and datasets (see “The pooling of p values via meta-analysis?”). (E) It facilitates causal inquiries and provides a metric to evaluate and determine the existence and strength of potential causation (see “The roles of the p value in causal inference, feature selection, and predictive modeling” for more details).

Certainly, there will be people who will still choose to believe, even given strong quantifiable evidence, that the Earth is not round or that a vaccine developed to deal with a virus is not useful. The p value, along with the probabilistic belief system it forms, however, provides a platform through which most people with a shared (probabilistic) belief system can compare, debate, or reproduce the findings (Figure 4). Whereas studies with large p values (especially in small sample studies) do not conclude that the findings yield no scientific insights, if one observes an extremely small p value in a study (for a given sample size) while others with similar settings do not, then this suggest that either there is something wrong with this study (therefore worth investigating) or something exciting is happening (also worth investigating). If small p values are observed consistently across samples and laboratories (and, especially, when the directions of the effects are also consistent), there is a stronger consensus that there may be something scientifically meaningful. It does not necessarily raise findings to the level of knowledge but brings it closer to it (or prevents bad results from being adopted into scientific understanding) through such explorations. Finally, there is no divorce between the p value and Bayesian evidence; when there are only data and no prior knowledge, one can rely on the hypothesis test and the p values to gain insights about the data, and when prior knowledge exists, one can use the prior to modify information gained from the observations and vice versa. Under “Hypothesis test in the Bayesian realm,” we show that the Bayesian evidence is, in essence, a compromise (or integration) between prior knowledge and data-driven knowledge.

Nevertheless, when making discussions, debates, or conclusions leveraging evidence derived from the p value-based hypothesis testing, one needs to remind oneself to consider the context. How were the samples collected, and are the samples representative? What is the sample size? How are the data aggregated (were samples spuriously combined; see “The hacking and misuse of the p value”)? Are p values consistent from sample to sample? And so on. See the use of the p value in context under “Some paradoxes and misuses of the p value.”

### The interpretation of the p value

#### The philosophy of the p value

In our view, a hypothesis testing framework links a population (e.g., a group of individuals), a statistical model, and probabilistic belief. Inspired by Roger Penrose’s three-world system linking the physical, mathematical, and mental worlds,^43^ the population has a property (e.g., the prevalence of a disease in the population) that is interesting to the investigators; the property is governed by a data-generating mechanism that is not yet well known or difficult to state explicitly; to gain insights about this particular property, one develops a hypothesis about the data-generating principle. To evaluate this hypothesis, one then draws a sample (via a proper statistical manner, such as randomization) and tests whether there is evidence for it. The hypothesis test produces a p value with which one assigns probabilistic belief about the property and decides whether to reject the hypothesis (Figures 2 and 3F).

### The roles of the p value in science

The p values must confront a few challenges. First, it may be possible that the sample property does not well represent the population property. Next, the unknown property of the population may not be well established using a statistical argument (e.g., a test done on a sample whose distribution violates the assumption of the test). Thus, the p value and the belief attached to it (to make any statement about the population property) via a hypothesis test may be inconsistent with the true (but unknown) population property.

In spite of criticisms, the p value has been of great interest to biological and medical scientists, clinicians, ecologists, economists, philosophers, and statisticians in its three-century-long history.^8^^,^^9^^,^^10^^,^^11^^,^^12^^,^^18^ Hypothesis testing and the p value form a knowledge-acquiring system that derives evidence from a sample; they also form an inferential system that throws probabilistic light on the population. There are, in general, four important roles the p value plays in scientific inquiries. First, it allows comparing and bridging decision-making outcomes regarding the same testing problem done on different studies and datasets. “Different individuals faced with the same testing problem may have different criteria of size. Experimenter I may be satisfied to reject hypothesis H using a test with size 0.05, whereas experimenter II insists on using 0.01. It is then possible that experimenter I rejects H, whereas experimenter II accepts H on the basis of the same outcome of an experiment. If the two experimenters can agree on a common test statistic, this difficulty may be overcome by reporting the outcome of the experiment in terms of the p value.” (See page 221 in Bickel and Doksum.^44^) Second, it supports evidence at a continuous (rather than binary) scale: “…the smaller the p value, the stronger the evidence for rejecting the null hypothesis. Hence, a p value reports the results of a test on a more continuous scale, rather than just the dichotomous decision ‘Accept the null hypothesis’ or ‘Reject the null hypothesis.’” (See page 397 in Casella and Berger.^45^) Third, it enables merging results from multiple studies and datasets. When different experiments produce various types of data, the p value can combine the evidence relating to a given hypothesis.^46^ This is the basis for “data fusion” and meta-analysis^47^ (see below for further discussion). Fourth, it facilitates causal inference, feature selection, and predictive modeling.

### The roles of the p value in causal inference, feature selection, and predictive modeling

Hypothesis tests and the p value make important contributions to causal studies, feature selection, and predictive modeling.

**Figure 5:**
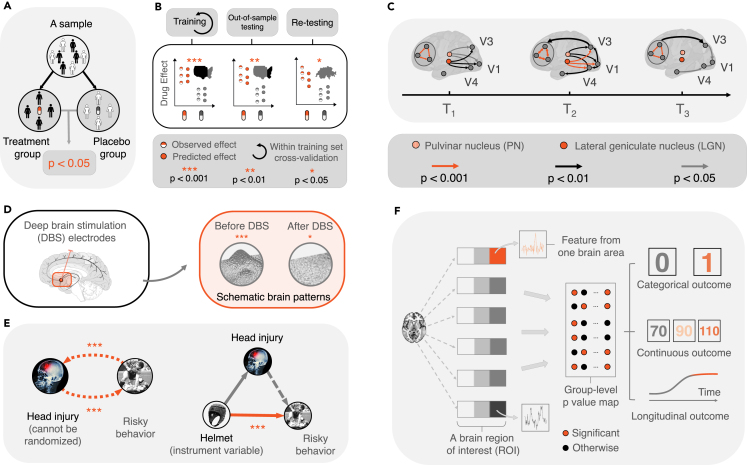
The roles of hypothesis testing and the p value in making causal inquiries, feature selection, and predictive modeling (A) Estimation of a causal effect. The average causal effect in a randomized study can be identified and quantified using the difference between the expected outcome of the treatment group and the control group and can subsequently be examined via a p value. (B) Out-of-sample test. The model performance or the causal effect estimated from one dataset, when not validated, may be exaggerated or overfit the dataset. Out-of-sample testing can, to a certain degree, alleviate overfitting by training the model using a subset of the data (left) and testing it in the remaining, previously unseen, data (center). Additional testing using data from another study or demographic distinctive sample may further support the generalization of the trained model and its suggested causal claims (right). The p value is critical to evaluate whether the tests are successful, thereby guarding their validity and efficacy. (C) Graphical causal reasoning. The directed arrows (called edges) indicate potential causation. The figure gives a schematic example of the potential directed causal flows in the brain when performing moving object recognition. When one views a moving object, areas in the visual cortex, including V1, V3, and V4, first receive input from the pulvinar nucleus (PN) and lateral geniculate nucleus (LGN) (left). Subsequently, V1 sends signals to V3 (which processes dynamic form recognition) and V4 (which processes color recognition), and through V3, sends information to the prefrontal cortex (center). Finally, there is reverse feedback from V3 and V4 to V1 (right). (D) Causal alternation. If altering the cause (while controlling for covariates) results in a change in the outcome, then it suggests that the stimuli cause the change in the outcome. The figure gives an example of deep brain stimulation (DBS), where, when applying DBS to a target brain region, the brain patterns of the area change accordingly, which then modifies (behavioral) symptoms. DBS is used in treating severe Parkinson's disease (PD). (E) The method of instrumental variable (IV). When directly altering causes or randomization is unavailable, one can consider the method of IV. Someone is interested in studying whether a head injury causes risky behavior. On the one hand, randomization or assigning a head injury is impossible; on the other hand, it could be argued that reverse causation, where risky behavior causes a head injury, is also possible. By using an IV (i.e., wearing a helmet), one can then study whether a head injury causes risky behavior. Suppose one assumes that wearing a helmet is unlikely to cause risky behavior (in the long term), and it is likely to reduce (the chance of getting) a head injury. If introducing wearing helmets reduces risky behavior (while controlling for all other variables, such as age and gender), then it suggests that wearing helmets reduces head injury, which reduces risky behavior. (F) The role of p values in feature selection and predictive modeling. From left to right: each box refers to a brain region; boxes with the same color but different hues indicate the same anatomical or functional brain area. Hypothesis testing between brain data and clinical (categorical, continuous, and longitudinal) outcomes yields a whole-brain p value map. Based on the p values, one can select features (biomarkers); the orange dots indicate selected (significant) features. These features, when coupled with estimated weights (not shown), can be used to predict categorical, continuous, or longitudinal outcomes in previously unseen subjects.^55^

First, the p value helps to estimate a causal effect (see Figure 5A). Suppose a researcher is interested in studying whether a levodopa-based drug is effective in treating Parkinson’s disease (PD). They need to compare the symptoms of a PD patient after taking the drug with those of the *same* (our emphasis) patient not taking the drug. Only one of the two is observable, and within-subject designs are not suitable because of carry-over effects. Using randomization, the Neyman-Rubin causal model (or the potential outcomes framework) shows that the average causal effect can be *identified* and estimated using the difference between the expected outcome of the treatment group and the expected outcome of the control group (without randomization, one cannot derive causal properties from two groups consisting of different individuals).^48^^,^^49^^,^^50^ There are times when randomization becomes impossible. For example, it is unethical to assign a group of 45-year-old healthy subjects to take a new levodopa-based drug to investigate whether the drug reduces one’s PD symptoms at 50. Additionally, there is likely another source, say, socioeconomic status (which may be related to the affordability of new drugs) or genetics (if there is a family history of PD, one may be more willing to take the drug), that is associated with taking the drug and developing PD at 50. Similarly, it would be difficult to estimate the effect of taking the drug on reducing PD symptoms by comparing the PD symptoms of an individual at 50 who had taken the drug with his or her PD symptoms at 50 had he or she not taken the drug. To solve these issues, propensity score matching (PSM) estimates the treatment effect by comparing the outcomes of the subjects under treatment (e.g., taking the drug) with a set of “matched” subjects without treatment (e.g., not having taken the drug).^51^^,^^52^^,^^53^^,^^54^ More concretely, one could first compute the propensity score of A taking the drug based on his or her gender, economic, social, genetic, and demographic background and choose an individual from a group of 50-year-olds who had not taken the drug but has a propensity score (of taking the drug during his or her younger years) closest to A’s. Then we can compare the PD symptoms between these two individuals and estimate the effect of taking the drug on reducing PD symptoms at age 50. By evaluating the p value, a hypothesis test can then examine whether, and, if so, to what extent, the drug effect from the treatment group is more significant than that of the control group.

Second, the p value facilitates out-of-sample testing. The p value is useful to verify whether evidence (e.g., hypothesis testing conclusions and model performance) discovered in a sample can be extrapolated to another independent sample (Figure 5B). For example, if one is interested in developing a model to select neural markers that can predict the severity of PD (say the Movement Disorder Society-Sponsored Revision of the Unified Parkinson's Disease Rating Scale [MDS-UPDRS] score), one can first fit the model on brain data obtained from a training sample of 70 people during model development. Subsequently, one can test whether the neural markers developed from the training data can predict the MDS-UPDRS scores in 30 previously unseen subjects without further modeling. The efficacy of the selected neural markers can be evaluated by comparing how well the predictions are made using a distance measure (e.g., Pearson correlation) and its p value. If significant, then one can say that the model fitted on the training set is reproducible (regarding the test set). Additionally, the p value can be used to test whether the model trained (and results obtained) from one study (including within-study training and testing) can be extrapolated to or reproduced in another dataset or study.^56^^,^^57^ Neither type of out-of-sample test, strictly speaking, examines *causation*; an out-of-sample study endorsed by a significant p value, however, reduces the likelihood of model overfitting. Although an overfit model suggests nothing about causation, a reproducible model does offer stronger evidence of association. In short, out-of-sample testing potentially yields more rigorous statistical claims about model performance and potential causal relationships between variables under investigation. Overall, when significant results are discovered from an experiment, it is useful to repeat the experiment to verify whether the result can be replicated or reproduced.^58^

Third, the p value is useful in graphical causal reasoning, whose modern development is based on Reichenbach’s macro statistical theory^59^ and Suppes’ probabilistic theory^60^ (interested readers could refer to the books edited by Sosa^61^ and Sosa and Tooley^62^ for a thorough review). Suppose one uses a graphical model to study how activities from brain region A may be causing those from region B (Figure 5C). One can perform a hypothesis test and use the p value to evaluate whether a significant directed edge exists from A to B (or from B to A).^63^^,^^64^^,^^65^

Fourth, the p value is useful to study causal alteration. It examines whether the modification of a hypothesized cause, results in a change of the hypothesized effect while fixing other potential causes (Figure 5D). For example, via transcranial magnetic stimulation (TMS), one can use a magnetic field coil to generate electric current, which modifies the magnetic field of a specific group of neurons in a small surface region of the brain.^66^^,^^67^ After controlling for confounds, one can perform a hypothesis test to examine whether there is a significant difference between the outcomes (e.g., human behavior) when these neurons are “on” with the outcomes when they are “off” and conclude, based on the p value, whether these neurons are responsible for the outcome change.

When a direct manipulation of the cause is impractical, the p value is useful when employing the method of instrumental variable (IV; Figure 5E).^68^ For example, head injury in rugby players may cause behavioral, emotional, and sensory changes (such as developing risky behavior, becoming irritable and angry, and having trouble with balance). A significant correlation between the severity of head injuries and changes in behavior, emotions, and sensation, however, does not conclude that the former causes the latter. On the contrary, having risky behavior and being irritable and angry may result in fights between players, whereas having a poor sense of balance may cause falling, both of which may result in head injuries. Furthermore, a head injury may first affect another variable, such as developing depression, which then affects the behavioral, emotional, and sensory changes. One cannot randomize individuals to receive a head injury but could relatively easily introduce an additional variable, or IV, that affects the chance of having a head injury but has no independent effect on the outcome (i.e., the behavioral, emotional, and sensory changes). More specifically, a suitable IV is one that is correlated with an endogenous explanatory variable, such as the severity of a head injury, but is not correlated with the error term (for example, in a regression), where an endogenous explanatory variable is a covariate that is correlated with the error term. A possible IV here is wearing helmets (in rugby union, players usually do not wear helmets), which may reduce the chance of having a head injury but does not directly affect the outcomes. If, after introducing the helmet, the behavioral, emotional, and sensory changes become insignificant, then one can conclude with more confidence that head injuries are the cause of changes. The p value helps to evaluate the effect size, strength, and direction of the causal effect of the IV.

Finally, hypothesis testing and p values are useful for feature selection and predictive modeling. Via hypothesis testing, one finds variables (or features) that are significantly associated with an outcome (for example, disease severity)—this may help to explain the outcome. It also helps out-of-sample predictions. Features are first selected during model development. Subsequently, one can couple the selected (significant) features with their trained weights to predict outcomes for previously unseen subjects^55^ (Figure 5F). In general, there are two ways to perform feature selection and predictive modeling. The first approach is to use a stepwise hypothesis test, such as a mass univariate analysis. During each step, a hypothesis test examines one feature and its association with an outcome to decide whether to admit or discard the feature (see multiple testing under “A note on multiple comparisons”). The selected features can then enter a predictive model for further training and testing. The second approach performs feature selection and prediction simultaneously, using statistical methods such as regularized models. The weights of less significant features are shrunk toward (or strictly to) zero, thereby removing these features. Out-of-sample prediction can then be made using the remaining features and their trained weights. One can then evaluate the validity of the chosen features by checking prediction performance and looking at their scientific or biological relevance.^55^

Certainly, there are other contributions that hypothesis testing and p values make to science, but it would be difficult to list every derivative. Although the applications may differ from one subject to another, the roles of the p value suggest that there are common merits it offers to general studies. We hope that our presentation may stir further discussion and that the ever-expanding statistical and scientific knowledge will one day allow us to formulate more universal statements about hypothesis testing and the p value.

### Some paradoxes and misuses of the p value

In this section, we discuss a few paradoxes and misuses of the p value. “The relationship between the p value, sample size, and power” makes enquiries into the relationships between the p value, sample size, and significance level in hypothesis testing and decision-making. “The hacking and misuse of the p value” presents common p hacking strategies in scientific studies. “Statistical significance (p < 0.0x) vs. clinical relevance” compares statistical significance and clinical relevance. “Big data and the p value” discusses the connection between the p value and big data. “Recommendations for avoiding misuses of the p value” and Table 1 summarize modest tips to deal with misuses and misinterpretations of the p value. “Making better use of the p value” suggests a pipeline for making potentially more effective use of the p value in scientific studies. We hope that our discussions and suggestions, by no means exhaustive, may improve the use of the p value to deliver more consistent and reproducible scientific discoveries.

#### The relationship between the *p value*, sample size, and power

Suppose a clinician wanted to test whether the prevalence of a disease was 10%. To do so, the clinician selected a sample of 10 individuals, found that two of the 10 had the disease, and used evidence from the sample (20% sample incident rate) to make inferences about the population prevalence. With p = 0.26, the hypothesis was not rejected.

The first paradox is that decisions made on the same effect size from data of different sample sizes may be inconsistent. For example, suppose we increased the sample size from 10 to 50, of which 10 had the disorder (the sample incident rate remained at 20%). This yielded a p value of 0.02. Although the new sample had the same (20%) incident rate, the null hypothesis was rejected under a significance level of 0.05. This test, however, would still fail to reject the null under a significance level of 0.005. Now consider an even larger sample of 100, of which 20 had the disease (the sample incident rate remained 20%), but the p value was 0.002. The hypothesis was rejected under 0.005.

**Figure 6:**
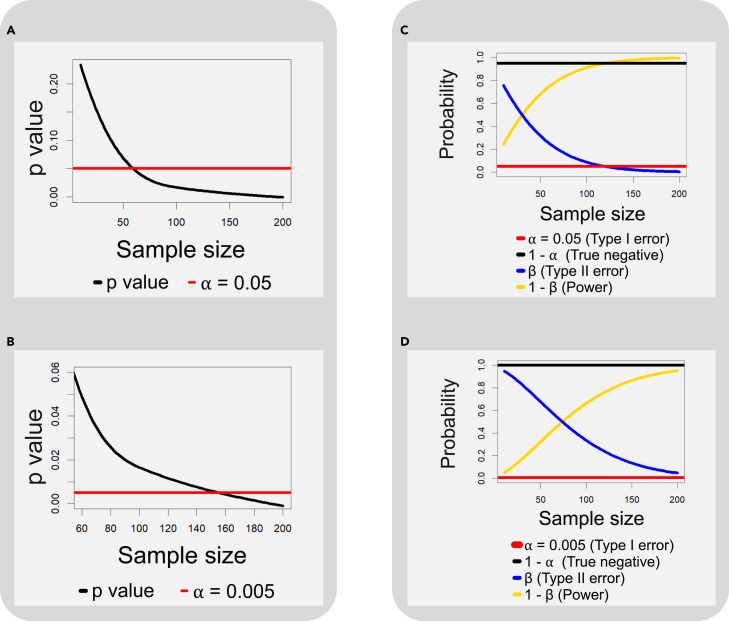
The paradoxes of the p value (A) The associations between the p value, the sample size, and the significance level. The figure shows that the p value goes down as the sample sizes increase. The paradox lies in that, given a particular significance level (say 0.05), one can increase the size of the sample to obtain a p value that is significant. (B) Even if the significance level is lowered (to, say, 0.005), one could keep increasing the sample size to obtain a significant p value. On the other hand, with a fixed sample size, one may adjust the significance level to “control” whether the result is significant. (C) The paradox between the p value, the sample size, and statistical power. A larger sample size may yield a more significant p value with a small effect size, but it also increases power. (D) Reducing the significance level (say, from 0.05 to 0.005) may produce more conservative testing results, but it reduces power. (A)–(D) demonstrate, from different perspectives, why the interpretation of the p value needs to be contextual.

**Figure 7:**
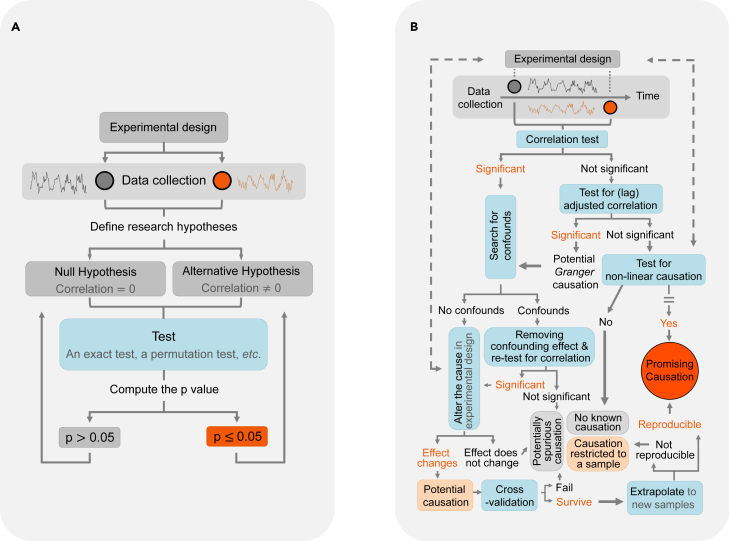
Making better use of the p value (A) A typical flowchart for conducting hypothesis-led testing of, for example, whether the correlation between two random variables is significantly different from zero. A significant correlation, however, does not equate causation. Note that this framework forms the first part of the flowchart in (B). (B) A more rigorous flowchart. We use the correlation test as an example, which can be replaced with other models or tests. It can also extend to cases involving more than two variables. For demonstration, we focus on testing linear causation and abbreviate the procedure for testing non-linear causation (which is marked with two parallel bars; interested readers can refer to Bai et al.^97^ and Hiemstra and Jones^98^). The illustration demonstrates that even simple analysis needs additional caution when causal inference and reproducibility are concerned. Such a flowchart, however, is not the only way to perform hypothesis testing; rather, we show that a more streamlined pipeline may help remove confounding effects, avoid overfitting, and facilitate reproducible research. A careful experimental design, appropriate data processing, and contextual scientific interpretation (not shown) are also important.

Generally, the p value decreases monotonically as the sample size increases, a phenomenon perhaps first observed by Berkson^69^ (see an example in Figure 6). Thus, a hypothetically aggressive scientist may attempt to “hack” the p value by adding more subjects to the study or by repeating significance tests. To avoid this, one may consider sample size and effect size during experimental plans. For example, in clinical trials, a phase II study is first done to determine effect size and population variation, and this information is then used to power a phase III study to ensure collecting enough samples to detect the difference. Indeed, given unlimited resources, most people may prefer studies with very large sample sizes because they feel larger sample studies are more reliable than smaller trials. Here, we do not advocate against large-sample studies (which have many advantages, as we see below); rather, we argue that one should treat the p value contextually and avoid being that aggressive scientist^70^ (see suggested guidelines in Table 1 and Figure 7).

**Table 1:** A brief summary of common misinterpretation and misuse of the p values and recommendations^22^^,^^23^^,^^94^^,^^95^^,^^96^

Misuse and misconception of the p value	Recommendations
(1) Scientific conclusions and decisions are based on whether a p value is less than a specific threshold.	Observing a p value less than a threshold (*e*.*g*.*,* 0.05) alone does not, and should not, endorse a binary scientific conclusion. This point is crucial when the p value is close to the threshold. For example, neither a rejection of a null hypothesis when p = 0.045 nor a failure to reject one when p = 0.055 offers conclusive evidence regarding the null; such close calls need further analyses, such as cross-validation, test-retest (e.g., permutation and bootstrap tests), and out-of-sample extrapolation. By further evidence, it means that, when reporting a p value is mandatory (e.g., by a journal, consortium, or funding organization), reproducing a significant p value is highly recommended. For example, when a significant p value is discovered in a training sample, check whether an independent testing sample also yields a significant p value. If modeling is concerned, verify whether fitted parameters obtained from a discovery sample can be extrapolated to a previously unseen testing sample. Extrapolation here means applying a trained model to new test data and examining whether meaningful prediction can be made without further model fitting on the test data.
(2) *p hacking* (e.g., conducting several statistical tests and only reporting those that pass the threshold).	Instead of “hacking” the p, (re)evaluate whether the experimental design is appropriate (e.g., is the design balanced; is the sampling randomized?), data collection is appropriate, the data processing is rigorous, the model is suitable, and all assumptions are met. If multiple statistical tests are conducted on the same data or several tests are done on different datasets, then report all analyses and their p values. In these cases, if a single p value is required (by a journal, consortium, or funding organization), then conduct a proper meta-analysis to combine p values (see “The pooling of p values via meta-analysis?”).
(3) The value 0.05 is the “gold-standard” significance level.	We cannot offer a strong recommendation for a “gold-standard” significance level. The number 0.05 was coined by Fisher for convenience (see “The rise of the p value”). In general, we suggest that, when data are too small to be split into a training set and a test set, use a conservative significance level for confirmative discovery (e.g., 0.05 is more conservative than 0.1). Whenever possible, replicate the result in a new sample. For large data that can be split into a training set and a test set, consider a conservative significance level (e.g., 0.005) for training and a relatively more liberal one (e.g., 0.05) for out-of-sample prediction.
(4) The p value measures the probability that the research hypothesis is true. The p value measures the probability that observed data are due to chance.	The p value measures the tail probability of the distribution of a test statistic; it makes a statement about whether observed data supports a hypothetical research explanation. It does not give a statement about the explanation.
(5a) I have a very large sample. (5b) I have conducted a hypothesis test and obtained a very small p value. (5c) Thus, the result must be significant.	The p value is sensitive to sample size and variability in the sample. A very large sample size with a very small effect size can yield a significant p value. Such results may offer little inference in scientific studies and are likely to be irreproducible.^93^ When facing large sample sizes, one may consider a data-driven approach instead (see point 6). If, however, a small but significant effect size is reproducible, the finding *may* still shed light on basic science, but it needs to be contextual (see point 1). In biomedical studies, one could begin with a statistical statement; for example, “the difference was statistically significant,” followed by an additional statement on the clinical significance, using the effect size and their directions.
(6) Scientific discovery must be accompanied by hypothesis testing and a p value.	They are standard or popular ways to extract scientific evidence, but they are not the only ways. Depending on the specific scientific question, prior insights, and observed data, a few alternative approaches are sometimes more suitable and feasible than hypothesis testing. For example, scientists can also report confidence, credibility, or prediction intervals to indicate effect size and direction. If scientists have prior knowledge about the problem, then they could consider Bayesian evidence. There are other measurements for evidence, such as the likelihood ratio or Bayes factor (see supplemental information). Finally, one could consider approaches based on decision theory and FDRs.

For further reading, please see references 22, 23, and 94–96.^22^^,^^23^^,^^94^^,^^95^^,^^96^

The second paradox arises because of the relationship between the sample size, the p value, and power. To see this, let’s return to the example above. On the one hand, adding more data may appear as *p hacking*, but it improves power. Under the same significance level (say 0.05), the type II error decreases as the sample size goes up; as a result, the power increases. On the other hand, a stringent significance level is not always beneficial: comparing Figures 6C and 6D, a test with a more stringent significance level yields less power, and this is true for every sample size.

Taken together, although the incidence in the three samples (n=10,50, and 100) was the same, the hypothesis testing results were different. In other words, for each (lower) significance level, when the sample incidence rate was relatively stable, it was possible to obtain a significant p value by increasing the sample size, thereby “hacking” the test. This highlights that the interpretation of a p value needs to be contextual. Moreover, when designing experiments and conducting hypothesis testing, there is a compromise to make, one that considers balancing the sample size, significance level, and power.

To summarize:

(1) The p value-based hypothesis testing is sample size dependent.
(2) Lowering the threshold *alone* may make rejecting a null hypothesis more difficult, but one may increase the sample size to “hack” the p value.
(3) Increasing the sample size may yield a more significant, but not necessarily meaningful, p value (see “The hacking and misuse of the p value”), but it increases power. Reducing the significance level (say from 0.05 to 0.005) may produce more conservative testing results but reduces power.
(4) The interpretation of the p value needs to be contextual, accounting for the experimental design, model specification, sample size, significance level, desired power, and the scientific question.

#### The hacking and misuse of the p value

In this section, we provide examples of common *p hacking* strategies.^6^^,^^71^^,^^72^^,^^73^ We hope our exploration could help better identify their various disguises and avoid the misuses of the p value, whether done consciously or innocently, in practice. For theoretical detection of *p hacking*, one may refer to work by Elliott et al.^74^

In the following, we summarize six types of common mistakes. In brief, the first is regarding inflating the sample size (see “The relationship between the p value, sample size, and power”). The second is about omitting a part of the data. For example, one performs a hypothesis or runs a model on a subset of the data that yields significant results or removes “outliers” containing meaningful signals. The third is regarding mistreating multiple variables, either during multiple comparisons, feature selection, or predictive modeling. For example, one does not correct for multiple comparisons (to reduce, for example, spurious correlations), or searches for and adds more variables, after performing a hypothesis test or modeling to boost significance. The fourth is not having a well-formed hypothesis or applying a test or model to data that do not actually test the outlined hypothesis, such as pseudo-replication. The fifth is about misusing hypothesis tests, such as applying the t test to highly skewed data. Last but perhaps not least is selective reporting: running several experiments, fitting several models, or applying various transformations to the data but only reporting the significant one(s). Certainly, here we mainly focus on errors that are somewhat disguised or may be made by an oversight. We do not discuss strategies such as rounding the decimals or forging data, which are superficial or downright unscientific.

##### *p hacking*

We define *p hacking* as taking inappropriate steps, whether consciously or innocently, to obtain significant p value(s) in science. Compared with misuses discussed below, which are not completely unfounded but not ideal, *p hacking* is, in general, inappropriate statistical practice, and one should strive to avoid it.

##### Inflating sample size

Under “The relationship between the p value, sample size, and power,” we have shown how, by increasing the sample size, one can “obtain” a small enough p value.

##### Pseudo-replication

Related to inflating sample size is pseudo-replication. In scientific studies in general, and biological studies in particular, replications help distinguish and quantify variations because of biological (or treatment) replicates and repeated measurements (technical replicates that may be due to devices). Yet, caution is needed. For example, one can take blood samples from 10 potential patients and check, using a cell analyzer, whether the average CD4 count is significantly below a threshold. One can also take three blood samples for each individual and evaluate whether, and, if so, to what extent, there are technical variations because of the cell analyzer. Suppose none of the patients has significantly lower CD4 counts than a threshold; by combining the 30 replicates (3 technical replicates times 10 subjects), one may observe, and erroneously report, that the average CD4 count (among these 10 subjects) is significantly less than the threshold.

##### Significant but spurious relationships

When an algorithm selects features by looking for variables that are significantly associated with the outcome, not all the selected features are necessarily meaningfully linked with the outcome. This is because, when the number of features is quite large, some of them are likely to be spuriously associated with and predictive of the outcome. A similar phenomenon may appear in multiple comparisons; when one makes a large number of (pairwise) comparisons, it is likely that some of the pairs are spuriously significantly related. Additionally, when one performs several hypothesis tests, it may be erroneous to obtain one significant p value by joining p values, (especially) from different test statistics (e.g., t, chi-square, and Wilcoxon tests) because the joint distributions of the test statistics are unknown; even when the same type of test statistics are involved (e.g., t tests for multiple endpoints), the correlations between the test statistics are often unknown. In these cases, one can use stepwise, gatekeeping, and group sequential multiple test procedures (MTPs) to control the family-wise error rate (FWER)^75^ (see “A note on multiple comparisons”).

##### Data fishing (by studying a subset of the whole data)

Statistical analysis aims to extract information from the (entire) data. Data fishing, however, uses data analysis to find useful information *the way one wants*. A common way of data fishing is to perform the otherwise standard analysis on a (selected) part of the data and argue that the findings hold for the whole sample and therefore extend to the population. For example, suppose one wants to examine the efficacy of a new drug. A useful way is to run a randomized control study with half of the randomized sample taking the drug and the other half taking a placebo and then evaluate the difference between the two means. Suppose the means are not statistically significant; one way to make (proper) further investigation (to check whether there are any neglected considerations) is to break the sample into different age and/or gender groups and study the drug effect for each group. Another is to match subjects (via, for example, PSM). But if, instead, one finds a subset of the treatment sample (who took the drug) that shows positive effects and then compares them with a matched pair who took the placebo and finds that the difference between these sub-samples is significant and argues that the drug is effective, one is fishing for information (to show that the drug is effective). Certainly, there are more “discrete” ways of sub-sampling, but our message remains: the results are likely biased and not reproducible because one omits people in the treatment group who show little to no drug effect.

##### Removing meaningful “outliers”

Outlier detection (or novelty detection) is an important, oftentimes necessary part when handling real-world data. Outliers are either driven by noise or measurement errors or given rise because they come from a mechanism different from the one under investigation. For normally distributed data with outliers, removing true outliers may make the data distribution more normal, thereby satisfying the assumption of several prominent statistical models; for other distributions, removing outliers may reduce bias (for example, the estimated parameter will be biased if estimated on outliers and the rest of the data). Not all data that fall outside of the common (not necessarily normal) distribution, however, are outliers. Sometimes they may be unique cases with new scientific insights. For example, in HIV studies, some individuals have innate immunity to the HIV virus; removing them may delay the discovery of mutation of the gene encoding CCR5. Other times, they may be from a less represented subpopulation. For example, for patients with diabetes, about 90% have type 2 diabetes and 10% have type 1 or gestational diabetes. For a model trained on a randomly selected diabetes sample, if a small percentage of type 1 diabetic patients are considered outliers, its parameters may fail to capture type 1- or gestational diabetic-specific information. In general, when the removal of outliers may not be optimal, one may consider robust estimators.^76^

##### Selective reporting

Selective reporting comes in several forms. The most common ones are running several experiments but reporting only the significant one(s) or trying different statistical tests and reporting one(s) that give significant results. One of the most embarrassing moments in a statistician’s career is perhaps when one asks whether there is a statistical test that will give significant results. Certainly, the persons who asked the question were, oftentimes, not ill intentioned. But one needs to reflect on statistical education, which statistical experts and those who apply statistics should work together to improve. Indeed, one can investigate data using several types of hypothesis tests or models so long as one does not hide all insignificant ones, and even when only a proportion of the tests gives significant results, it still suggests that there may be meaningful information worth investigating further. It is when one cherry-picks one test (or model) that yields significant findings (and/or only reports the significant one(s)) that concerns arise. Certainly, some tests require assumptions (such as the normality assumption in a t test), and if the data meet the assumption, then one can start with a particular test. Oftentimes, real data do not meet all statistical assumptions (normality, independence, etc.); even when they do, several tests (and models) may be suitable. In these cases, we encourage our readers to report findings from all tests performed (and models run) and conduct model comparisons to provide themselves (and their audience) with a holistic view of the data and the process leading to the argued results.

##### Misuses of p values

Practices such as model selection, data transformation, and variable selection are common practices in statistics. But when done inappropriately, they may yield errors.

##### Trying different models

Model comparison is critical in statistical science. One proposes a (statistical or machine learning) model with the hope of optimally describing a system (or phenomenon). Up until that point, one knows, at best, a few probable models that may be suitable candidates, but no one knows which may work the best. Therefore, one runs (or develops) several candidate models and sees which fits the data best. To do so, one runs model comparisons and cross-validations and finds an optimal model that minimizes errors or maximizes the likelihood. The chief point here is that the candidate models should be laid out before model comparison and drawing conclusions. If one tries *sequentially* different models until one finds a model that gives the “desired” results, then one may bring about soft *p hacking*.

##### Transformation

Another standard statistical technique is data transformation. For example, via generalized linear models, one transforms the outcome (via a link function) so that the relationship between the transformed outcome and the variables is linear. It is simple because the (transformed) relationship is linear, and it helps explanation (e.g., logit link function can be interpreted as log odds) and prediction. However, it becomes problematic when one transforms one set (or column) of data but not the other sets (columns). A more stealthy practice is when one applies an arbitrary (arbitrarily complex) function so that the model fits well (e.g., gives good prediction) for a set of (e.g., biological or medical) data. Despite advances in explainable artificial intelligence (XAI), some of the black-box models may fall into these territories. The predictions are good, but the explanation is feeble; we do not know why the hidden layers and activation functions work or how they facilitate scientific explanation. Certainly, it is useful to design black-box models: if one’s goal is prediction, then an accurate black-box model may suffice. But if one wants to gain insights into a biological or physical phenomenon, then the transformation made into data via hidden layers and various activation functions does not seem to, as of yet, deliver significant biological or medical intuition.

##### Increasing the number of variables

A study may consist of a single, several, or large number of variables. Suitable univariate, multivariate, and high-dimensional statistical methods are developed to deal with each of these cases. Yet, suppose the original experiment considered q (q ≥ 1) variables, but none yielded significant results, and one subsequently added more variables hoping to find significant ones; this is improper. Indeed, if one has realized, after running the model, that one forgets to include important variables, such as age and gender, then one needs to re-fit the model (although one perhaps needs to make a reflection). In general, however, one should include and fix all variables of scientific relevance or interest during the experiment design (i.e., before performing hypothesis testing, variable selection, and model fitting). One should avoid adding new variables after statistical analyses to get more significant results. If one must append additional variables, then one needs to document the rationale and steps.

##### Misuse of tests and models

Parametric tests are useful to detect differences between groups, but they typically require distributional assumptions. For example, Li et al.^77^ investigated why DESeq2 and edgeR, two popular methods to identify differentially expressed genes (DEGs) under two (e.g., normal vs. disease) conditions, had many false-positive discoveries and suggested that the poor result was in part due to violation of the negative binomial distribution assumed by both methods. Similar to the misuse of tests, model misspecification would also yield erroneous p values. For example, when the relationship between a set of features (exposures) and an outcome is non-linear, the p values generated using linear models are likely to be erroneous, and so are the identified features.

### Statistical significance (p < 0.0x) vs. clinical relevance

The paradoxes of the p value raise the need to distinguish statistical significance and clinical relevance. First, a significant p value may not equate to clinical relevance. When employing hypothesis tests in clinical studies, a p value that is greater than or equal to the significance level (e.g., 0.05) only indicates a lack of evidence to reject the null hypothesis; it is not equivalent to “no difference between groups.” A statistically significant effect need not to be indicative of a large or meaningful effect size. Second, ignoring a statistically non-significant clinical finding from a sample with high variation or a small sample size may neglect useful information or yield publication biases.^78^^,^^79^

Mayo and Spanos^80^ used the concept of post-data severity evaluation to explain how p value-based decision-making can cause the fallacies of acceptance (when no evidence against *H_0_* is mistreated as evidence for it given low test power and small sample size in detecting sizable discrepancies) and rejection (when the evidence against $H_{0}$ is interpreted as evidence for a particular $H_{1}$ given high test power and large sample size for trivial discrepancies).^80^ The fallacy of rejections concerns the dangerous practice to conflate statistical significance with substantive (or clinical, for most medical research questions) significance or, to be more specific, to conflate the statistical alternative with a substantive theory.^80^ The null and alternative hypotheses under the Neyman and Pearson framework must exhaust the parameter space of a given statistical model and thus only allow the alternative hypothesis to be deduced upon the null being rejected, but not based on a substantive theory or knowledge.

Hypothesis testing-based p value alone may oversimplify a clinical question or provide insufficient information regarding the clinical (trial) results. In clinical trials and drug development, “clinical significance,” which may be a cardinal element in driving treatment decisions,^81^ typically refers to the magnitude of the actual treatment effects; it suggests whether the results of, say, a trial, can impact current medical practice. Information on minimal clinically important differences (MCIDs) or minimal important changes (MICs) needs to be discussed beforehand based on prior knowledge or experiments. Model interpretation in clinical studies, therefore, needs combined expertise from statisticians, clinicians, and general scientists. In addition to evaluating a p value, it is useful to take the effect size and the direction of the effect into consideration.^82^ Suggestions under “Recommendations for avoiding misuses of the p value” may be useful in this regard.

Another way is to report the p value and a confidence interval (CI).^83^ Although there is a mathematical duality between CI and hypothesis testing, the CIs are less vulnerable to the large n problem and contain, arguably, more information than p values.^84^ By presenting CI along with the p value, one may steer away from purely seeking statistical significance and into considering statistical significance in light of clinical relevance. The advantage of including the CI is that CI (1) reports results directly on the scale of the data, (2) provides the direction and strength of the effects, (3) partly implies sample size and variability through its width,^82^^,^^85^^,^^86^ and (4) avoids the problem of sharp dichotomy (e.g., rejecting null at p = 0.0499 but failing to do so at p = 0.0501).^34^

#### Big data and the p value

Bigger data provide a larger platform to make scientific enquiries and, properly treated, may produce more consistent conclusions.^55^^,^^87^ In the following, we will present a few perspectives regarding the relationship between the p value and big data.

First, big data may introduce big errors. Large-scale data, such as magnetic resonance imaging (MRI) data, may contain large-scale noise. For example, in fMRI data, multiple sources of noise, such as scanner-related noise, including thermal noise and scanner instability noise, noise because of head motion and physiology, HRF model errors, and noise because of different sites, can corrupt the true signals.^55^ There are three ways to mitigate this issue. First, one can aim to reduce noise by, for example, improving data acquisition, pre-processing, and de-noising procedures before performing hypothesis testing. Second, scientists who consider a massive number of comparisons can improve reproducibility via cross-site and cross-study analyses and impose a very strict significance level (e.g., $5\times 10^{-8}$ for geneticists; $3\times 10^{-3}$ and $3\times 10^{-7}$, respectively, for “evidence of a particle” and for “discovery of a particle” in physics).^88^^,^^89^^,^^90^ Third, even with extensive replication and strong signals, one may still observe false discoveries because of confounding variables or other biases. Therefore, in addition to designing stringent test pipelines (e.g., Figure 7), integrating, and reproducing evidence, it is important to improve statistical thinking, teaching, and interdisciplinary training.^91^

A second problem with big data is the increasing likelihood of obtaining spurious findings. Consider a hypothesis test to investigate the relationships between 500 brain edges and individual creativity scores. Among the 500 edges under consideration, it is likely that a few of them will be *spuriously* associated with the outcome. This may introduce an erroneous scientific conclusion that these edges are underpinning creativity.

Third, a small effect may appear significant, although not necessarily meaningful, when studying big data. Empirically, a correlation of 0.1 in a sample of 500 has a p value around 0.025; a correlation of 0.01 in a sample of 100,000 has a p value around 0.002. The former is significant at α=0.05 and the latter at α=0.005, but the p values in these cases may offer little insight. In psychological and sociological investigations involving very large numbers of subjects, it is regularly found that almost all correlations or differences between means are statistically significant”.^92^ In clinical trials and pathological studies, a small but significant effect size may not offer much clinical inference and is difficult to interpret and reproduce.^93^

#### Recommendations for avoiding misuses of the p value

Here, we summarize recommendations for a few common misinterpretations and misuses of the p value.^22^^,^^23^^,^^94^^,^^95^^,^^96^ Before proceeding, let us ask a few questions.

(1) Should scientific conclusions be solely based on whether a p value is less than a specific threshold? Is *post hoc* scientific interpretation based on the p value justified?
(2) How could we prevent “*p hacking*” (for example, conducting several statistical tests and only reporting those that pass the threshold or adding subjects to existing studies to lower the p value in scientific discoveries)?
(3) Many studies report results when observing a p value smaller than 0.05, 0.01, or 0.005. But is 0.05, 0.01, or 0.005 an optimal bar?
(4) Does the p value measure the probability that the research hypothesis is true? Or does it measure the probability that observed data are due to chance?
(5) Does obtaining a very small p value from hypothesis testing using a very large sample provide conclusive evidence?
(6) Must scientific discovery always be accompanied by a hypothesis test and a p value? Are there alternative statistical approaches?

In Table 1, we attempt to answer these questions and present a collection of recommendations from the statistical and scientific communities with our minor comments. Under “Making better use of the p value” and in Figure 7, we use a flowchart to depict our suggestion on how to potentially make better use of the p value in hypothesis testing.

#### Making better use of the p value

Through our explorations, one may see that it is difficult to suggest an optimal sample size, significance level, or power with which everyone agrees. A compromise, however, can perhaps be made by suggesting a streamlined pipeline for conducting hypothesis testing aimed at improving reproducibility in scientific studies (Figure 7). One can see that even a seemingly simple associative analysis requires extra caution. We highlight that the interpretation of the p value is contextual. We need to interpret the p value along with, perhaps never independent of, the research (experimental) design, hypothesis, model and its assumptions, and prior evidence. Finally, it is important to improve statistical thinking and interdisciplinary training integrating statistical concepts and scientific insights.^91^

### Hypothesis test in the Bayesian realm

Comparing two different types of evidence, Bayesian evidence and the p value, is like comparing two belief systems. Indeed, one colleague has nicely summarized the discrepancy as follows: “I have always found the comparison between p values and posterior tail areas very puzzling because the p value is defined as a tail area where the value of the sample changes, but the posterior tail area varies over different values of theta (the unknown parameter). How are these two comparable unless we want to compare eggs with sausages?” Here, for completion purposes, and as the discussion and debate between Bayesian evidence and p value persist, we add this section to show an alternative way to gather knowledge via the Bayesian lens.^13^^,^^14^^,^^99^^,^^100^^,^^101^^,^^102^^,^^103^^,^^104^^,^^105^^,^^106^

Unlike the p value, which is determined by the observations and the statistical model $M_{\theta }\left(x\right)$ (i.e., completely data driven), Bayesian evidence depends not only on the observations and the model but also on *a priori* knowledge. In other words, if one has a strong prior (a very large precision relative to the likelihood), then no matter how much information the data contain, the posterior parameters are chiefly dictated by the prior. The hypothesis testing outcomes are, consequently, chiefly determined by the density function of the prior. On the other hand, a weak prior surrenders to data; the posterior parameters, therefore, are closer to the maximum likelihood estimators (MLEs) of the likelihood. Consequently, the hypothesis testing outcomes may be chiefly determined by the likelihood function. When a uniform prior (perhaps the most extreme case of a non-informative prior) and a Gaussian likelihood are employed, it is relatively easy to see that the p value and the Bayesian provide the same information. To see these points more concretely, let’s consider an example.

Suppose we have some prior knowledge about a parameter $\mu ∼N\left(\mu_{\pi },\sigma_{\pi }^{2}\right)$. The likelihood of drawing data ${x=(x}_{1},x_{2},\ldots ,x_{n})$ is $P\left(x|\mu \right)=\prod_{i=1}^{n}P\left(x_{i}|\mu \right)={\left(2\pi \sigma_{x}^{2}\right)}^{-\frac{n}{2}}\exp\left\{-\frac{1}{2{\sigma_{x}}^{2}}\sum_{i=1}^{n}{\left(x_{i}-\mu \right)}^{2}\right\}$. It follows that after seeing data x, the posterior distribution of $\mu |x∼N\left(\mu_{n},\sigma_{n}^{2}\right)$, where $\mu_{n}={\left(\frac{n}{\sigma_{x}^{2}}+\frac{1}{{\sigma_{\pi }}^{2}}\right)}^{-1}\left[\frac{n}{\sigma_{x}^{2}}\left(\frac{\sum_{i=1}^{n}x_{i}}{n}\right)+\frac{1}{{\sigma_{\pi }}^{2}}\mu_{\pi }\right]$, and $\sigma_{n}^{2}={\left(\frac{n}{\sigma_{x}^{2}}+\frac{1}{{\sigma_{\pi }}^{2}}\right)}^{-1}$. Suppose $\mu_{\pi }=90$ and ${\bar{x}}_{n}=110$, and we wish to examine two sets of hypotheses.

$${\left(S1\right):H}_{0}:\mu \leq 109\;\text{vs.}\;H_{1}:\mu >109\text{;}$$

$${\left(S2\right):H}_{0}:\mu \leq 111\;\text{vs.}\;H_{1}:\mu >111$$

Let’s consider scenarios that cover three fundamental relationships between the precision of the prior and that of the likelihood.

(1) The prior is more precise (with a smaller standard division) than the likelihood; i.e., $\sigma_{\pi }:\sigma_{x}=1:5$.
(2) The prior has similar precision as the likelihood; i.e., $\sigma_{\pi }:\sigma_{x}=5:5$.
(3) The prior is less precise than the likelihood; i.e., $\sigma_{\pi }:\sigma_{x}=5:1$.

**Figure 8:**
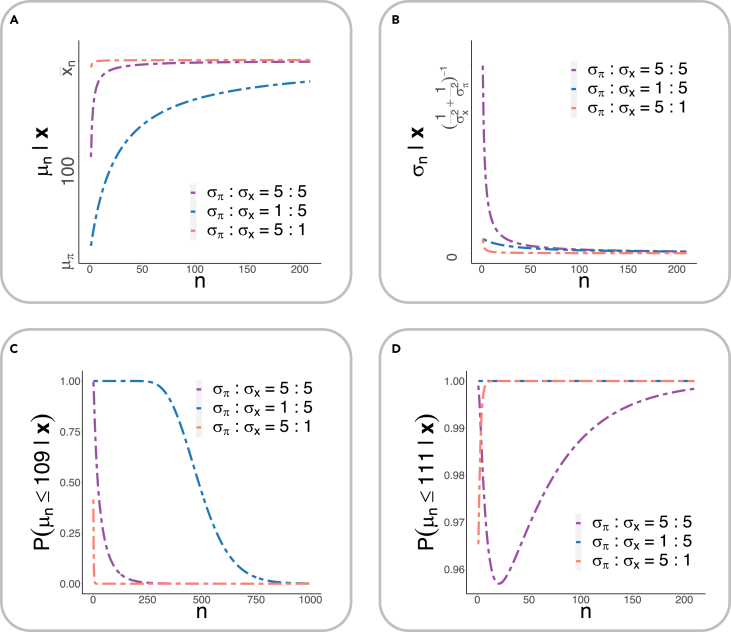
An illustration of Bayesian posterior evidence (A) The behavior of the posterior mean. (B) The behavior of the posterior standard deviation. (C) The behavior of posterior evidence for $H_{0}:\mu \leq 109$. (D) The behavior of posterior evidence for $H_{0}:\mu \leq 111$. See text for explanations.

In all scenarios (Figures 8A and 8B), the posterior mean approaches the mean of the likelihood function (which equals the MLE) as more data are gathered (i.e., as n increases), and the variance (which determines our confidence about the accuracy of the posterior estimate) decreases toward zero. The rate of convergence, however, differs across the three scenarios. When the prior is less precise than the likelihood (e.g., $\sigma_{\pi }:\sigma_{x}=5:1)$, the posterior mean converges to ${\bar{x}}_{n}$ (the MLE) rather quickly with smaller *a posteriori* variance, as the dominating information is provided by the data. When the prior is more precise than the likelihood (e.g., $\sigma_{\pi }:\sigma_{x}=1:5)$, the posterior mean is quite resistant to converging to ${\bar{x}}_{n}$ (the MLE) with a larger *a posteriori* variance; unless a lot more data are used, the posterior takes into significant consideration the prior. When the prior and the likelihood have similar precision (e.g., $\sigma_{\pi }:\sigma_{x}=5:5$), the convergence rate of the posterior mean is moderate.

The usefulness of Bayesian evidence lies in that it balances (or modulates) prior knowledge and knowledge gathered from the data (the likelihood).^106^ Let’s consider the first set of hypotheses (S1): $H_{0}:\mu \;\leq 109$ vs. $H_{1}:\mu \;>109$. The mean of the prior ($\mu_{\pi }=90$) is in favor of the null, but the likelihood is not (${\bar{x}}_{n}=110$). When the prior is not as precise as the likelihood (e.g., $\sigma_{\pi }:\sigma_{x}=5:1$), the posterior mean gives up supporting the null immediately after seeing the data from a distribution centered at 110 (the red line in Figure 8C). When the prior is more precise than the likelihood (e.g., $\sigma_{\pi }:\sigma_{x}=1:5$), however, it requires more data to convince the posterior mean (the blue line in Figure 8C).

Next, let us consider the second set of hypotheses (S2): $H_{0}:\mu \;\leq 111$ vs. $H_{1}:\mu \;>111$. The means of the prior ($\mu_{\pi }=90$) and the likelihood (${\bar{x}}_{n}=110$) are in favor of the null, and the prior is more in favor of the null than the likelihood. When the prior is more precise than the likelihood (e.g., $\sigma_{\pi }:\sigma_{x}=1:5$), the posterior went to support the null right after seeing a small number of data points (the blue line in Figure 8D). When the prior is not as precise as the likelihood (e.g., $\sigma_{\pi }:\sigma_{x}=5:1$), the posterior mean needed more data to support the null (the red line in Figure 8D). An interesting scenario occurs when the prior and the likelihood are not precise ($\sigma_{\pi }:\sigma_{x}=5:5$); in this case, it takes some battling, after seeing more data, to achieve consensus regarding supporting the null (the purple line in Figure 8D). See also an example of using the Bayes factor in model comparison in the supplemental information.

#### Bayesian posterior evidence vs. the p value

“Most nonspecialists interpret p precisely as $P\left(H_{0}|x\right)$ (see Diamond and Forrester;^101^ thereby committing the fallacy of the transposed conditional, our insertion), which only compounds the problem.”^13^

One chief difference between the p value and Bayesian evidence is that the former considers the parameter as an unknown but fixed value, and the latter considers the parameter as a random variable on which a probability distribution can be imposed. Naturally, it is difficult to compare an argument built on a fixed number with one built on a distribution. Yet, there is a special case.

For Bayesian evidence, when the prior brings in little information (i.e., a non-informative prior), it is possible that the Bayesian evidence may deliver the same amount of information (i.e., from the data alone) as the p value does. More vividly, let’s consider a case. Consider a uniform prior defined on the real line and Gaussian likelihood $x_{i}|\mu \;∼\;N\left(\mu ,\sigma_{x}^{2}\right)$. The posterior then is $\mu |x∼N\left(\mu_{n},\sigma_{n}^{2}\right)$, where $\mu_{n}={\bar{x}}_{n}$, and $\sigma_{n}^{2}=\frac{\sigma_{x}^{2}}{n}$. For a null hypothesis $H_{0}:\mu \leq \mu_{0}$, the Bayesian evidence is $P\left(\mu \leq \mu_{0}|x\right)=P\left(\frac{\mu -{\bar{x}}_{n}}{\frac{\sigma_{x}}{\sqrt{n}}}\leq \frac{\mu_{0}-{\bar{x}}_{n}}{\frac{\sigma_{x}}{\sqrt{n}}}|x\right)=\Phi \left(\frac{{\sqrt{n}(\mu_{0}-{\bar{x}}_{n}}_{})}{\sigma_{x}}\right)$, where Φ is the cumulative distribution function (CDF) for N(0,1). For the same hypothesis, the p value is $1-P\left(\Delta \left(X\right)>\Delta \left(x\right),\mu =\mu_{0}\right)=P\left(\Delta \left(X\right)\leq \frac{{\sqrt{n}(\mu_{0}-{\bar{x}}_{n}}_{})}{\sigma_{x}}\right)=\Phi \left(\frac{{\sqrt{n}(\mu_{0}-{\bar{x}}_{n}}_{})}{\sigma_{x}}\right)$. Thus, using a non-informative prior, the Bayesian evidence and the p value provide the same information.

Naturally, one would ask: which is more suitable for scientific studies? In general, we suggest that one may consider the Bayesian approach when one has some *a priori* belief about the parameter and considers the p value when one only has data without prior knowledge about them. There are a few general consensuses regarding the amount of evidence they provide. Because one- and two-sided hypothesis tests are the predominant practices in scientific expositions, we will focus on these two types of tests in the following. Readers who are interested in composite hypothesis tests could refer to Bayarri and Berger^99^ and Berger et al.^100^

1. For a two-sided (point null) test: the p value tends to overstate the evidence against the null;^13^^,^^102^^,^^104^ that is, the p value is smaller than the Bayesian posterior evidence.
2a. For a one-sided test: the p value can be approximately equal to the Bayesian posterior evidence.^107^
2b. For a one-sided test: one can construct an (improper) prior so that the p value and the Bayesian posterior evidence match.^108^
3a. For a one-sided test: for data following distribution with a monotone likelihood ratio that has unimodal density, symmetric about zero, or is normal $\left(0,\sigma^{2}\right)$, where $0<\;\sigma^{2}<\infty$, the p value is equal to $\inf\;P\left(H_{0}|x\right)$, where the infimum is taken over a class of priors.^14^
3b. For a one-sided test: for other distributions, the p value is greater than or equal to $\inf\;P\left(H_{0}|x\right)$, suggesting that the p value may be understating the evidence against the null.^14^
4. If a prior mass is concentrated at a point (or in a small interval), and the remainder is allowed to vary over the alternative hypothesis $H_{1}$ (in other words one has strong prior information), then there could be a (noticeable) discrepancy between the Bayesian posterior evidence and the p value (see examples in Casella and Berger^14^).

Taken together:

(1) For a two-sided test (e.g., testing whether the disease prevalence is not equal to 20%), the conclusions made using Bayesian evidence may be more conservative than using the p value.^13^^,^^102^^,^^104^
(2) For a one-sided test (e.g., testing whether the disease prevalence is above 20%), the two offer approximately the same evidence (and can be constructed to be equivalent).^107^^,^^108^
(3) When one has strong prior information about the null hypothesis, the Bayesian alternatives would favor the null.^14^
(4) When samples are large, small p values (see discussions above and Figure 6) almost systematically reject the null; the Bayesian alternatives may not.^109^
(5) One should be aware that, if different studies adopt different priors, then it would be problematic to compare findings between studies.^110^
(6) If one has a lot of (quality) data, then one may want to hear the opinions of the data (Figure 8).

#### The pooling of p values via meta-analysis?

The analysis of large-scale datasets has two attractive aims: information accumulation and commonality extraction.^55^

Information accumulation can be done by either increasing the size of a single dataset or combining different datasets. For the former, it can expand the sample size (by collecting more subjects), enrich temporal dynamics (by obtaining more longitudinal measurements for each subject), and augment spatial variability (by increasing the number of spatial features collected or areas measured for each subject). For the latter, it can compound heterogeneous samples, disease categories, or task paradigms. The p values obtained from large-scale datasets may more clearly suggest the difference between subpopulations (e.g., healthy versus disease, male versus female, individuals under various treatments, or stimuli versus controls) and identify the pathological-, gender-, treatment-, and task-specific phenotypes. Hypothesis testing and the p value obtained from repeated measurements help to delineate the longitudinal changes of the features, thereby potentially improving disease assessment over time and paving the way for longitudinal disease prediction and progression monitoring.^111^^,^^112^^,^^113^^,^^114^^,^^115^

Commonality extraction refers to obtaining converging evidence from multiple studies and datasets. Datasets obtained from different studies and experimental conditions may contain heterogeneous signals. They may also be subject to different degrees of systematic bias because of different experimental designs (e.g., a complete factorial design versus a fractional factorial design^116^), noises (such as head motion^117^), measurement errors (because of data aggregation under different paradigms and from different sites),^118^ missing data,^119^ and reporting bias (for example, only positive results are reported^120^). Consequently, data analysis results reported from mis-specified models^121^ or datasets obtained under different designs and conditions may provide different p values, thereby generating different, sometimes opposite conclusions.

Naturally, one would ask, how then can one obtain evidence from various studies and datasets? The meta-analysis (analysis of analyses) is a useful approach to integrate and extract evidence from large-scale heterogeneous datasets, reduce reporting bias, and draw potentially reliable conclusions by pooling multiple p values from different studies and datasets.

(1) Meta-analysis can integrate results from different studies. For example, Fisher’s and Pearson’s methods combine the p values obtained from multiple studies and datasets (Figure 4).^122^^,^^123^ When data are sparse (for example, when there are only a small number of features associated with an outcome in high-dimensional data), one can use Tippett’s minimum p value test,^124^ the higher criticism test,^125^ and the Berk-Jones test^126^ to improve power.^127^
(2) Meta-analysis may reduce bias. For example, when regions of interest have more liberal thresholds than others (such as in large-scale neuroimaging studies), the results are likely biased toward these regions. Meta-analyzing functional and structural data across multiple large-scale studies can reduce bias and improve power.^128^^,^^129^ First, peak coordinates (e.g., the brain regions where the differences between healthy and disease are the highest) are combined with t-statistic maps (each t-statistic map can be plotted to the brain space where regions with large *t* values indicate activation). Second, statistical maps and effect-sizes maps are recreated. Finally, individual maps are combined according to intra-study variance (i.e., studies with large sample sizes and/or lower error contribute more) and inter-study heterogeneity (i.e., studies with large variances contribute less).
(3) Meta-analysis can examine whether discoveries are reproducible. Like a leave-one-subject-out cross-validation, it can perform a leave-one-study-out cross-validation. For example, it first compares the estimate (e.g., mean activation of a brain lesion) from one study to the summarized estimate from the remaining (n-1, where n is the number of total studies) studies and then iterates the process and judges, via the p value, whether the conclusions made across the studies are reliable and reproducible.

Today, it is increasingly common to see studies considering and balancing information accumulation and commonality extraction. For example, a committee of researchers may organize several study groups conducting multiple experiments and gathering data at different locations under various conditions, a good practice that has already been adopted in clinical trials (multicenter studies), to seek converging evidence that may address a common scientific question.

When performing a meta-analysis of p values, however, one needs to be cautious when the p values are obtained from different test statistics. First, meta-analysis may be *inappropriate* in practice unless all p values share (approximately) the same statistical context (including the model, framing of hypotheses, sample size, etc.).^121^ When p values come from different test statistics (e.g., t, chi-square, and Wilcoxon tests) or the same test statistics with unknown correlations, one can adopt MTPs to control the FWER.^75^ When the test statistics or p values are correlated (for example, in genome-wide association studies [GWASs], some SNPs can be highly correlated; in brain imaging studies, values from some voxels in a brain can be highly correlated), one can combine p values in these settings leveraging the Cauchy distribution.^127^ Alternatively, one can combine p values from multiple tests by scaling up their harmonic mean by a factor^123^ or use conformal inference.^130^

## A note on multiple comparisons

When performing multiple tests, the probability of observing significant results, purely due to chance, rises. More specifically, for N independent tests at the α level, the expected number of false positives is Nα, and the probability of making at least one type I error, also called the FWER, is $1-{\left(1-\alpha \right)}^{N}$. One of the simplest and perhaps most widely used methods to address this (multiple testing) issue is the Bonferroni correction, which rejects p values that are less than α/N to control the FWER at level α. An alternative method to control the FWER is the Bonferroni-Holm method, a step-down procedure that adjusts significance thresholds less conservatively; it is less straightforward to implement but is uniformly more powerful than the Bonferroni correction in detecting true effects (see supplemental information for details).

In genomics studies, testing hundreds of thousands of genes for potential association with a treatment is a routine task; in brain imaging studies, one needs to examine hundreds of thousands of voxels for potential effects from a stimulus. The number of type II errors increases sharply with N. Indeed, with the rise of high-throughput data, the FWER has been criticized for being too conservative, causing large type II errors. To address this challenge, Benjamini and Hochberg^131^ introduced the false discovery rate (FDR) and imposed the empirical Bayes perspective.^132^ By ranking p values, they introduced a method that effectively controls the FDR, which represents the expected proportion of false discoveries, or type I errors (see supplemental information). Instead of controlling the probability of making one type I error at level α, the method ensures that the expected proportion of type I errors among the total number of significant results is less than α×100%. This permits a less conservative test and a decrease in the number of false negatives. Stated differently, it allows a small proportion of type I errors to significantly decrease the number of type II errors.

Naturally, one might wonder when p values should be corrected and, if so, which error type (FWER or FDR) should be controlled. There is no simple answer to this question. The correction of p values depends again on the context and on the purpose for conducting the multiple tests. In general, the aims of the tests fall into three categories: (1) to test pre-specified hypotheses that are not related, (2) to test one null hypothesis through testing of multiple sub-hypotheses (e.g., given a null hypothesis that states that a gene cluster is not related to treatment; if one p value is significant, the null hypothesis would be deemed rejected), and (3) feature selection (e.g., differential expression analysis). Accordingly, (1) requires no correction; (2) requires FWER control, such as Bonferroni-Holm; and (3) requires FDR corrections.

Nevertheless, some are concerned about increasing the number of false negatives when applying corrections, which may undermine the tests’ ability for new discoveries. Some are also concerned about the efficacy of the routinely used correction devices—whether they effectively control the number of false positives. Additionally, some may be uncomfortable to consider trade-offs involved in using correction techniques when dealing with real data. These concerns are not unjustified, and several authors have raised issues that may impact the efficacy of correction methods: (1) model misspecification, (2) the presence of outliers, and (3) the dependence between tests. For example, for (1) and (2), model misspecification and the presence of outliers may raise the number of false discoveries in genomics.^77^ For (3), multiple testing methods often assume that the tests are independent; however, in practice, where dependence appears (for example, co-regulated genes and spatially correlated blood-oxygen-level-dependent [BOLD] signals), such assumptions may lead to under- or overcorrection.^133^ While one can reduce the errors because of (1) and (2) by carefully selecting models, checking assumptions, and evaluating outliers, there are no universally agreed treatments for dealing with (3). Yet, since the introduction of the FDR, correction under dependence has been actively investigated, with new methods being vigorously developed. One popular way to address the dependence issue is to consider common factors.^134^^,^^135^ Using this approach, the dependence between the test statistics will be significantly reduced, and standard correction can be applied. The problem of dependence between tests, however, cannot be fully addressed by simply studying p values, as classical procedures do. Rather, it requires effective modeling of the interdependencies among features, a task particularly challenging in fields such as biology because of the high dimensionality and intricate nature of biological processes. A beginning, however, can perhaps be made by considering a multiple-comparisons framework that rests on statistical methods but allows adjustment based on scientific (e.g., biological) insights.

### Conclusion

In this review, we aimed to discuss the roles, challenges, and merits of the p value in hypothesis testing. We first outlined the roles the p value plays in scientific studies and discussed the associations between the p value, sample size, significance level, and statistical power. Subsequently, we presented common *p hacking* strategies as well as misuses and misinterpretations of the p value, accompanied by modest recommendations. To complement our discussion, we compared statistical significance and clinical relevance. Additionally, we presented the Bayesian alternatives of seeking evidence. Finally, we discussed the potential usefulness and challenges of performing meta-analyses to integrate p values from multiple studies and datasets and included a note on multiple comparisons.

To summarize, hypothesis testing and the p value form a useful decision-making system; they provide a common, simple rule that guides experimenters in evaluating and comparing findings via p values; they help to examine test outcomes on a continuous scale; they enable, when appropriately done, results integration from multiple studies and datasets; and they facilitate causal enquires, feature selection, and predictive modeling. Today, they are supporting scientific enquires to test the relationship between group, idiosyncratic, genetic, and environmental features; the difference between outcomes from multiple geographical units (such as crop yields from different fields) and biological units (such as patterns from different brain areas); how external stimuli and environmental factors affect genetic organizations and biological characteristics (such as heart rate and brain signals); how these patterns underpin human behavior; and how their irregularity may lead to malfunction and illness.

We believe that the p value will continue to play important roles in hypothesis-testing-based scientific enquiries, whether in its current form or modified formulations. We also believe that there will be a continued effort to seek more rational ways to extract knowledge from data and a more holistic interpretation of statistical and scientific evidence.

Because the employment of hypothesis testing and p values is and will for the foreseeable future remain one of the standard practices in scientific enquiries, a beginning can perhaps be made by improving our understanding of its roles, weaknesses, misuses, and merits. Our discussions highlight that its applications and interpretation must be contextual, considering the scientific question, experimental design, statistical power, effect size, prior knowledge, and reproducibility. Finally, if some of our explorations have brought you insights into your current and future studies, then we have received the utmost reward.

## Data and code availability

All data are available in the main text.

## Supplemental information

Document S1. Supplemental information and Figures S1 and S2

Document S2. Article plus supplemental information
