# Supplementary material for: The roles, challenges, and merits of the p value
Source: Patterns (N Y). 2023 Dec 8;4(12):100878. doi: 10.1016/j.patter.2023.100878 (PMC10724370; doi:10.1016/j.patter.2023.100878)
Supplement: Document S1. Supplemental information and Figures S1 and S2 [file mmc1.pdf]

Patterns, Volume *4*

Supplemental information

The roles, challenges, and merits of the p value

Oliver Y. Chén, Julien S. Bodelet, Raúl G. Saraiva, Huy Phan, Junrui Di, Guy Nagels, Tom Schwantje, Hengyi Cao, Jiangtao Gou, Jenna M. Reinen, Bin Xiong, Bangdong Zhi, Xiaojun Wang, and Maarten de Vos

Supplementary material: The roles, challenges, and merits of the p value

Oliver Y. Chén, Julien S. Bodelet, Raúl G. Saraiva, Huy Phan, Junrui Di, Guy Nagels, Tom Schwantje, Hengyi Cao, Jiangtao Gou, Jenna M. Reinen, Bin Xiong, Bangdong Zhi, Xiaojun Wang, and Maarten De Vos

Supplementary material for Subsection "The rise of the p value".

The fundamental goal. The fundamental goal of performing hypothesis testing is to derive evidence from the observed data, say $\boldsymbol{x}_0 \coloneqq (x_1, x_2, \ldots, x_n)$, to uncover the data generating mechanism $\mathcal{M}$ that yields $\boldsymbol{x}_0$. More precisely, one wants to learn about the properties of a parameter $\boldsymbol{\theta} \in \Theta \subset \mathbb{R}^m$ underpinning $\mathcal{M}$. Denote the true (but unknown) parameter as $\boldsymbol{\theta}^*$. The data-generating mechanism can be expressed as (Spanos 1986):

$$\mathcal{M}^*(\boldsymbol{x}) = \{f(\boldsymbol{x};\ \boldsymbol{\theta}^*)\}, \quad \boldsymbol{x} \in \mathbb{R}_{\boldsymbol{X}}^n$$

where $f(\boldsymbol{x};\ \boldsymbol{\theta}^{)}\}$ indicates the joint distribution of $\boldsymbol{x}$ with fixed value $\boldsymbol{\theta}^*$ and $\mathbb{R}_{\boldsymbol{X}}^{\boldsymbol{n}}$ denotes the sample space.

Since $\boldsymbol{\theta}^*$ is unknown (thus $\mathcal{M}^*(\boldsymbol{x})$ unknown), one needs a (realistic) statistical model $\mathcal{M}_{\boldsymbol{\theta}}(\boldsymbol{x})$ (see below) to learn about the true data generating mechanism by estimating the parameter from observed data $\boldsymbol{x}_0$. This can be summarized as $\boldsymbol{\theta}^* \underset{\mathcal{M}_{\boldsymbol{\theta}}(x)}{\overset{\mathcal{M}^*(x)}{\rightleftarrows}} \boldsymbol{x}_0$, where the top arrow represents the data generating mechanism and the bottom arrows indicate parameter evaluation via a statistical model.

(2) The statistical model. A hypothesis test uses a statistical model to learn about the true value of $\boldsymbol{\theta}$. A general model is:

$$\mathcal{M}_{\boldsymbol{\theta}}(\boldsymbol{x}) = \{f(\boldsymbol{x};\boldsymbol{\theta}), \boldsymbol{\theta} \in \Theta \subset \mathbb{R}^m\}, \quad \boldsymbol{x} \in \mathbb{R}_{\boldsymbol{X}}^n$$

where $f(\boldsymbol{x};\boldsymbol{\theta})$ denotes the joint distribution of $\boldsymbol{x}$ for any given parameter $\boldsymbol{\theta}$ in parameter space Θ and $n > m$.

Thus, the model $\mathcal{M}_{\boldsymbol{\theta}}(\boldsymbol{x})$ provides a vehicle to perform the hypothesis test to evaluate the choices of $\boldsymbol{\theta}$ in Θ to find an estimate close to $\boldsymbol{\theta}^*$. Critical to this process are probabilistic modelling assumptions, such as the identically distributed assumption, that underline $\mathcal{M}_{\boldsymbol{\theta}}(\boldsymbol{x})$. Any violation of the assumptions may yield misleading inferences regarding the parameter.

Under the pre-data view, two useful test statistics are:

- $\Delta_1(\boldsymbol{X}) = \frac{\sqrt{n}(\bar{X}_n - \mu_0)}{\sigma} \overset{\mu=\mu_0}{\sim} N(0,1)$.
- $\Delta_2(\boldsymbol{X}) = \frac{\sqrt{n}(\bar{X}_n - \mu_0)}{\sigma} \overset{\mu=\mu_1}{\sim} N(\eta,1)$, where $\eta = \frac{\sqrt{n}(\mu_1 - \mu_0)}{\sigma}$, for any $\mu_1 > \mu_0$

Let $c_1(\alpha) = \{\boldsymbol{x}$☹$\Delta(\boldsymbol{X}) > c_\alpha)\}$ be the rejection region. One can define the p value, Type I error (or α), Type II error (or β), and power (or $1 - \beta$) as follows.

p value:	$p(\boldsymbol{x}_0) = \mathbb{P}(\Delta_1(\boldsymbol{X}) > \Delta(\boldsymbol{x}_0); \mu = \mu_0)$.
Type I error:	$\alpha = \mathbb{P}(\Delta_1(\boldsymbol{X}) > c_\alpha; \mu = \mu_0)$.
Type II error:	$\beta \coloneqq 1 - \mathbb{P}(\Delta_2(\boldsymbol{X}) > c_\alpha; \mu = \mu_1)$.
Power:	$\mathbb{P}(\Delta_2(\boldsymbol{X}) > c_\alpha; \mu = \mu_1) = 1 - \beta$.

Notice that the probability of a Type II error cannot generally be computed because it depends on the population mean which is unknown. It can be computed, however, for given values of mean, standard deviation, and sample size.

Remark 1. The parameter is an unknown constant not a random variable in frequentist statistics. In the frequentist view of hypothesis testing, the parameter is considered as an unknown constant; not a random variable[1] (see (Spanos 2010)).

Similarly, the p value, the significance level (α), and power ($1-\beta$) also do not involve conditioning in frequentist statistics.

Remark 2. The parameter is a random variable in Bayesian statistics. What if we have some prior information about the parameter θ? For example, we have some (say weak) information about θ, and would like to perform a test to examine whether the data support the null hypothesis, say $H_0: \theta \leq \theta_0$? Said differently, we have some (distributional) information about θ, with which one could already (without seeing the data) form a degree of brief about the hypothesis by evaluating $\mathbb{P}(\theta \leq \theta_0)$. Then, after seeing data $\boldsymbol{x}$, would our brief about θ be changed?

Formally, suppose we have some prior knowledge that $\theta \sim N(\theta_\pi, \sigma_\pi^2)$. The likelihood of drawing data $\boldsymbol{x} = (x_1, x_2, \dots, x_n)$ is $\mathbb{P}(\boldsymbol{x}|\theta_x) = \prod_{i=1}^n \mathbb{P}(x_i|\theta_x) = (2\pi\sigma_x^2)^{-\frac{n}{2}} \exp\left\{-\frac{1}{2\sigma_x{}^2}\sum_{i=1}^n (x_i - \theta_x)^2\right\}$. It follows that after seeing data $\boldsymbol{x}$, the posterior distribution of $\theta|\boldsymbol{x} \sim N(\theta_n, \sigma_n^2)$, where $\theta_n = (\frac{n}{\sigma_x^2} + \frac{1}{\sigma_\pi{}^2})^{-1}\left[\frac{n}{\sigma_x^2}\left(\frac{\sum_{i=1}^n x_i}{n}\right) + \frac{1}{\sigma_\pi{}^2}\theta_\pi\right]$, and $\sigma_n^2 = \left(\frac{n}{\sigma_x^2} + \frac{1}{\sigma_\pi{}^2}\right)^{-1}$. Thus, the evidence for H_0 after seeing data is $\mathbb{P}(\theta \leq \theta_0|\boldsymbol{x}) = \mathbb{P}\left(\frac{\theta-\theta_n}{\sigma_n} \leq \frac{\theta_0-\theta_n}{\sigma_n}|\boldsymbol{x}\right) = \Phi\left(\frac{\theta_0-\theta_n}{\sigma_n}\right)$, where Φ is the CDF for $N(0,1)$.

Remark 3. The p value represents post-data evidence and is arguably inappropriate in a two-sided test. Type I error, Type II error, and power are *pre-data* probabilities (or evidence) (Spanos 2013). In other words, they do not involve observations $\boldsymbol{x} = (x_1, x_2, \dots, x_n)$. Practically, one sets the experimental conditions (for example, by choosing a specific sample size and the experimental mechanism during a clinic trial) such that the experiment yields pre-specified levels of Type I error, Type II error, and power. As such, these probabilities are embedded in the experiential design before the data have ever been seen.

Supplementary material for Section "A brief history of the p value".

A comparison between the Fisher's hypothesis test and the Newman-Pearson test

---

[1] To see this, suppose we can write the conditional density of data $\boldsymbol{x}$ given θ,

$$f(\boldsymbol{x}|\theta = \vartheta) = \frac{f(\boldsymbol{x}, \theta = \vartheta)}{\int f(\boldsymbol{x}, \theta = \vartheta) d\boldsymbol{x}}$$

where $f(\boldsymbol{x}, \theta = \vartheta)$ is a joint density, $\boldsymbol{x} \in \mathbb{R}_X^n$, and θ is a parameter underlying the statistical model $\mathcal{M}_\theta(\boldsymbol{x})$. In frequentist statistic, θ is some constant that either lays in the null parameter space $\Theta_0 \subset \mathbb{R}^m$ or the alternative parameter space $\Theta_1 \subset \mathbb{R}^m \backslash \Theta_0$ (see **Fig.** 1 c). Thus, the joint density $f(\boldsymbol{x}, \theta = \vartheta)$ makes no probabilistic sense (*i.e.*, is not variable with θ).

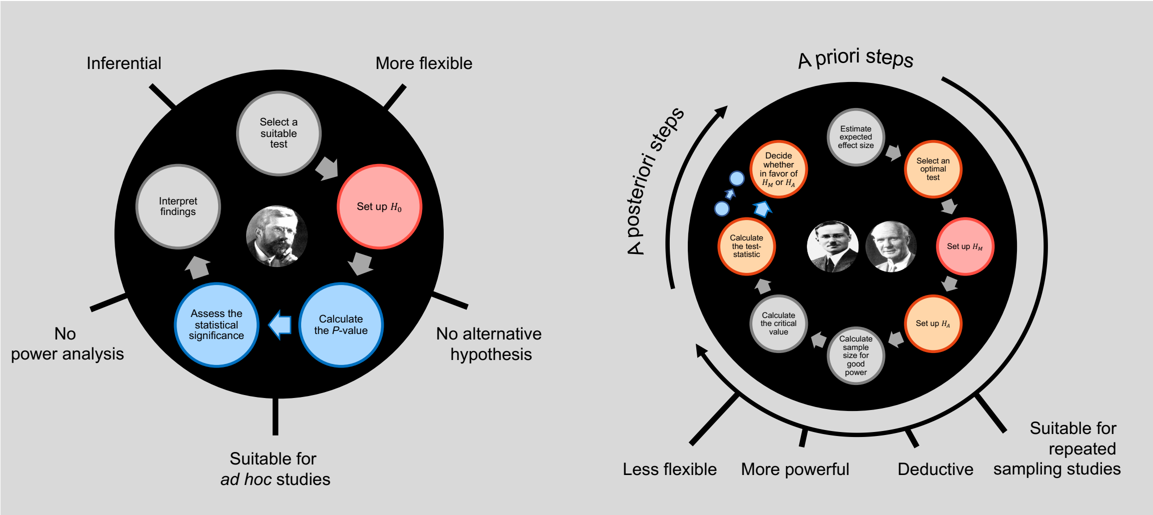

Fig. S1. A comparison between the Fisher's hypothesis test and the Newman-Pearson test, and how they constitute the null hypothesis significance test (NHST). Left: The Fisher's test by R.A. Fisher. It contains five main steps, following the order clockwise. Compared to the Newman-Pearson test, Fisher's test is more flexible, suitable for *ad hoc* studies, inferential, but it does not have an alternative hypothesis nor perform power analysis. **Right: The Newman-Person test** by Jerzy Neyman and Egon Pearson. It consists of eight main steps, following the order clockwise, where the first six steps are done a priori, and the last two steps a posteriori. Compared to Fisher's test, it is more powerful, deductive, and suitable for repeated sampling studies, but is less flexible. The null hypothesis significance test is a hybrid of the two; it follows the NP-test procedurally and Fisher philosophically (Cortina and Dunlap 1997; Hubbard 2004; Johnstone et al. 1986; Perezgonzalez 2015; Spielman 1978). Specifically, its mandatory steps consist of the steps highlighted in orange in the NP test, with the main hypothesis H_M replaced by H_o, and the p value calculation and significance assessment from the Fisher's test (highlighted in blue) added.

Supplementary material for Section "Hypothesis test in the Bayesian realm".

An Example: The *Bayes factor* in model comparison

It is not always necessary to report the Bayesian evidence in the context of posterior probability. One can also report the ratio of the posteriors of two hypotheses. A useful application is to perform a model comparison. Suppose an epidemiologist is interested in investigating whether the incidence rate of a disease is at 20% (H_1), or at 10% (H_2).

More concretely, suppose H_1 and H_2 are two hypothesized models parameterized by θ_1 and θ_2, respectively. The *Bayes factor* (see (Kass and Raftery 1995) for a comprehensive review), or K, is written as:

$$K = \frac{P(x|H_1)}{P(x|H_2)} = \frac{\int P(\theta_1|H_1)P(x|\theta_1,\ H_1)d\theta_1}{\int P(\theta_2|H_2)P(x|\theta_2, H_2)d\theta_2} = \frac{P(H_1|x)}{P(H_2|x)} \times \frac{P(H_2)}{P(H_1)} \quad (3)$$

where x stands for the data. Note that when the priors $P(H_1)$ and $P(H_2)$ are equal, the *Bayes factor* reduces to $K = \frac{P(H_1|x)}{P(H_2|x)}$, thus degenerating to a *likelihood ratio test*.

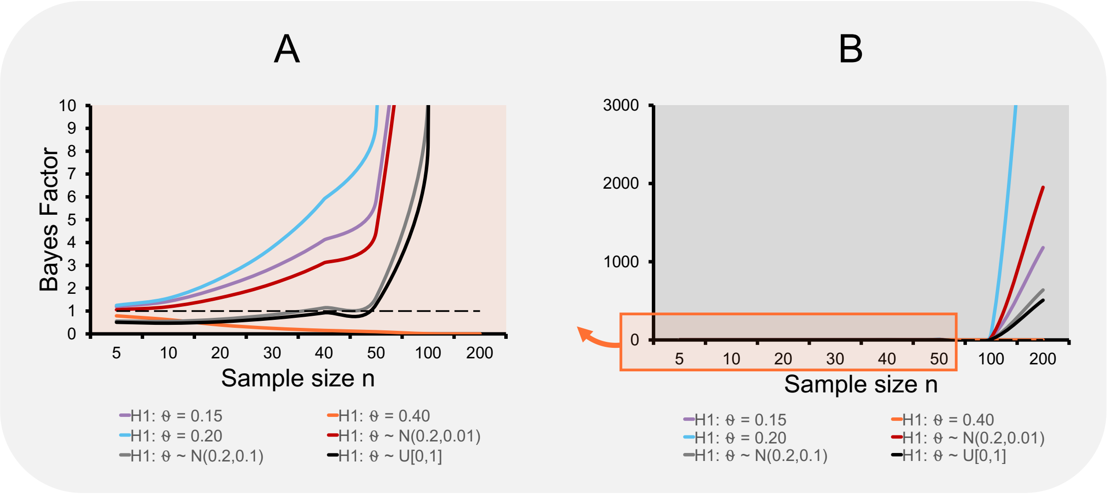

Fig. S2. An illustration of *Bayes factors* in model comparison. Consider an experiment comparing two models H_1 and H_2. For simplicity, the sample incidence rate was fixed at 0.2, no matter of sample size (that is, 8 for a sample of 40, and 20 for a sample of 100). Figure (a) is the zoomed-in snapshot of the orange box in Figure (b). **Figure (A)** shows how the *Bayes factor* changes when the sample size is smaller than 100; **figure (B)** shows how the *Bayes factor* behaves when the sample size is larger than 100. The experiment considered six candidate models for H_1 with the prevalence parameterized as follows: (1) from a uniform distribution or $\theta_1 \sim U[0,1]$; (2) 15%, or $\theta_1 = 0.15$; (3) 40%, or $\theta_1 = 0.40$; (4) 20%, or $\theta_1 = 0.20$ (which is the maximum likelihood estimator (MLE)); (5) from a normal distribution or $\theta_1 \sim N(0.2, 0.01)$, which can be considered as the MLE plus a small noise; and (6) from a normal distribution or $\theta_1 \sim N(0.2, 0.1)$, which can be considered as the MLE contaminated by a large noise, say, due to sampling error. The alternative model H_2 had a parameter $\theta_2 = 0.10$. The H_1 whose hypothesized parameter equalled the sample incident yielded the largest *Bayes factor*. In other words, the maximum likelihood estimator or MLE (in this case 0.2) achieved the optimal *Bayes factor*. The results also showed that the farther a hypothesized parameter departed from the MLE (*e.g.*, $\theta_1 = 0.40$ is farther from 0.2 than $\theta_1 = 0.15$), the smaller the *Bayes factor* (or evidence); this was true no matter of sample size; but the larger the sample size, the stronger the evidence. When the sample size was small, the model with $\theta_1 \sim N(0.2, 0.01)$ (namely, the MLE plus some Gaussian noise $N(0, 0.01)$) underperformed the model with $\theta_1 = 0.15$, indicating the noise had contaminated the evidence. With a larger sample size, the former outperformed the latter, indicating the signals from large-scale data had overcome the noise.

In words, the *Bayes factor* compares how likely the data are generated from model 1 (H_1) as compared to model 2 (H_2); hence the larger the K, the stronger evidence the data support H_1 over H_2. To see it more concretely, suppose the epidemiologist wanted to test the prevalence of a certain type of disorder. The epidemiologist came up with six candidate models (H_1) to test against an alternative model which assumed the prevalence was at 10% (namely H_2 considered a parameter $\theta_2 = 0.10$). The six candidate models considered their parameters as follows: (1) from a uniform distribution or $\theta_1 \sim U[0,1]$; (2) 15%, or $\theta_1 = 0.15$; (3) 40%, or $\theta_1 = 0.40$; (4) 20%, or $\theta_1 = 0.20$ (which is the maximum likelihood estimator (MLE)); (5) from a normal distribution or $\theta_1 \sim N(0.2, 0.01)$, which can be considered as the MLE plus a small noise; and (6) from a normal distribution or $\theta_1 \sim N(0.2, 0.1)$, which can be considered as the MLE contaminated by a large noise, say, due to sampling error.

The epidemiologist considered several samples of sizes 5, 10, 20, 30, 40, 50, 100, and 200. For comparison, suppose that the true incident rates were all at 20%; namely for each sample, there were, respectively, 1, 2, 4, 6, 8, 10, 20, and 40 patients. Using Equation (3), the *Bayes factors* for each test are calculated and presented in **Fig. S2**.

There are four messages we can draw from the simulation studies, from which one could peer into the general behaviour of the *Bayes factor*.

(a) When the hypothesis (in H_1) is close to the truth (20%), the *Bayes factor* uniformly supports H_1 over H_2 (as the *Bayes factor* is larger than 1 no matter the sample size).

(b) When the hypothesis (in H_1) is far from the truth, the *Bayes factor* uniformly opposes H_1 over H_2 (as the *Bayes factor* is no larger than 1 no matter the sample size).

(c) The larger the sample size, the stronger evidence the *Bayes factor* provides for supporting (or opposing) H_1.

(d) The *Bayes factor* accounts for prior information and uncertainties in the model. For example, when prior information about θ_1 is close to the truth (20%), the *Bayes factor* strongly supports H_1; when the prior is contaminated by some noise (as in $N(0.2, 0.01)$ and $N(0.2, 0.1)$), the *Bayes factor* becomes smaller, and the more noise found in the prior the smaller the *Bayes factor*. When there is uncertainty (as in a uniform distribution), the small sample size would support H_2 (namely $\theta_2 = 0.1$); when the sample size becomes sufficiently large, the *Bayes factor* learns (from the data) that it is increasingly unlikely that the data correspond to a model (H_2) where $\theta_2 = 0.1$.

Supplementary material for Section "A note on multiple comparisons".

To understand multiple comparison problems, assume a series of N tests with p values $p_1, p_2, \dots, p_N$. Under the null hypothesis, each p value is uniformly distributed, and thus, if a significance threshold α is used, the probability of obtaining a significant result is α. When performing N tests, at the level α, the chance of making at least one Type I error is $1 - (1 - \alpha)^N$ if independence is assumed (that is one minus the chance of making no errors). The Bonferroni correction controls the FWER by rejecting null hypotheses for $p_i \leq \alpha/N$ without any dependence assumption. Indeed, using Boole's inequality, we have $P\left(\bigcup_{j=1}^{N} p_j \leq \alpha/N)\right) \leq \sum_{j=1}^{N} P\left(p_j \leq \frac{\alpha}{N}\right) = \alpha$. The Bonferroni approach is sensible in traditional scientific fields, where N is usually small. For example, when performing five tests at level 5%, one should perform each individual test at level 1%. When testing $N = 10{,}000$ genes, the individual threshold, however, becomes 0.0005%. The Bonferroni correction, therefore, is sacrificing the Type II error, that is, the number of false negatives. More accurate FWER methods include Holm and Hochberg's methods based on the ordering of p values, $p_{(1)} \leq p_{(2)} \leq \cdots \leq p_{(N)}$, both of which reject p values lower than a threshold. The Holm and Hochberg are, respectively, a step-down approach, with a threshold computed as $\min\left(i: p_{(i)} > \alpha/(N + 1 - i)\right)$ and a step-up approach, with a threshold computed as $\max\left(i: p_{(i)} \leq \alpha/(N + 1 - i)\right)$. Controlling the FWER, however, comes with the cost of the increased number of false negatives as the number of tests increases.

Benjamini and Hochberg proposed a linear step-up procedure also based on the ordered p values. Specifically, given a desired FDR level q, they compute a threshold value $k = \max\,(i: p_{(i)} \leq qi/N)$ and reject all hypotheses with p values below this threshold. They showed that when the test statistics are independent, the procedure effectively controls the FDR at level $FDR \leq q$.

References:

Cortina, J. M., and Dunlap, W. P. (1997), "On the logic and purpose of significance testing," *Psychological Methods*, **2**, 161–172.

Hubbard, R. (2004), "Alphabet soup: Blurring the distinctions between p's and a's in psychological research," *Theory & Psychology*, **14**, 295–327.

Johnstone, D. J., Barnard, G. A., and Lindley, D. V. (1986), "Tests of significance in theory and practice," *Journal of the Royal Statistical Society: Series D (The Statistician)*, **35**, 491–498.

Kass, R. E., and Raftery, A. E. (1995), "Bayes factors," *Journal of the American Statistical Association*, **90**, 773–795.

Perezgonzalez, J. D. (2015), "Fisher, Neyman-Pearson or NHST? A tutorial for teaching data testing," *Frontiers in Psychology*, **6**, 223.

Spanos, A. (1986), *Statistical foundations of econometric modelling*. Cambridge University Press, Cambridge, UK.

Spanos, A. (2010), "Is frequentist testing vulnerable to the base-rate fallacy," *Philosophy of Science*, **77**, 565–583.

Spanos, A. (2013), "Who should be afraid of the Jeffreys-Lindley paradox," *Philosophy of Science*, **80**, 73–93.

Spielman, S. (1978), "Statistical dogma and the logic of significance testing," *Philosophy of Science*, **45**, 120–135.
